# 16S rRNA-based metagenomics insights into the microbial diversity and functional attributes of soils from the rhizosphere of selected C4 crops of farms in Mpumalanga and Limpopo provinces, South Africa

**DOI:** 10.1371/journal.pone.0347776

**Published:** 2026-06-15

**Authors:** Mamonokane Olga Diale-Makhongela, Tiisetso Mpai, Francina Lebogang Bopape, Prudence Mtsweni, Adeola Salawu-Rotimi, Nemera Geleta Shargie, Abe Shegro Gerrano, Liesl Morey, Bongani Kubheka, Ahmed Idris Hassen

**Affiliations:** 1 Agricultural Research Council, Plant Health and Protection (ARC-PHP), Queenwood, Pretoria, South Africa; 2 Inqaba Biotechnological Industries, Muckleneuk, Pretoria, South Africa; 3 Agricultural Research Council – Grain Crops, Potchefstroom, South Africa; 4 Agricultural Research Council – Vegetables, Industrial and Medicinal Plants, Pretoria, South Africa; 5 Food Security and Safety Niche Area and Crop Science Department, Faculty of Natural and Agricultural Sciences, North-West University, Mmabatho, South Africa; 6 Agricultural Research Council, Biometry Unit, Central Office, Hatfield, Pretoria, South Africa; 7 Department of Plant and Soil Science, Faculty of Science, Engineering and Agriculture, University of Venda, Thohoyandou, Limpopo, South Africa; Universidade de Coimbra, PORTUGAL

## Abstract

The rhizosphere serves as a hub for a variety of microorganisms that are highly beneficial to crop production and improvement of soil health. However, intensive farming practices including utilization of agrochemicals can cause a decline in microbial diversity that could severely compromise soil health and crop productivity. Here we investigated the taxonomic abundance and functional diversity of the microbial communities of sorghum and pearl millet rhizosphere soil samples from sixteen farms in Mpumalanga and Limpopo Provinces of South Africa. Soil samples were collected at the rhizosphere of sorghum and pearl millet crops and pooled into 34 samples. The soil samples were used for 16S rRNA amplicon sequencing analysis, soil physicochemical properties, and community-level physiological profiles. The results indicated that carbon utilization was highest in the majority of soil samples from Jane Furse, which also demonstrated greater microbial richness. The 16S rRNA amplicon sequencing analysis provides insight into the relative abundance of soil microbial communities, where at phylum level Planctomycetes, Proteobacteria, and Actinobacteria were the most predominant in all farms, but their relative abundances varied. Our results revealed that physicochemical properties could affect microbial abundance and diversity. The distance-based redundancy analysis (dbRDA) explained 46.8% of the variation in the soil bacterial community structure, with Mn, Fe, NO₃⁻-N, and Ca identified as the key soil physicochemical variables shaping community composition. Thus, this study may contribute to advancing sustainable agricultural practices by providing baseline data that may inform future bioinoculant development.

## Introduction

Nowadays, agriculture is facing a major challenge in increasing crop production due to abiotic stresses [1]. Climate change has intensified the impact of drought, salinity, and heat, making them the primary constraints on crop growth and a major threat to global food security [2]. Most arable lands are prone to one or more abiotic stresses, which affect crop yields by 60% on average [3,4]. Alternatively, extensive use of agrochemicals, irregular rainfall patterns and intensive farming, such as tillage and crop management, can alter the soil’s physicochemical properties, including pH, electrical conductivity, and organic matter content, ultimately affecting the soil microbiome and fertility [5,6]. Thus, developing more efficient and sustainable farming practices is essential to protect agricultural soils and ensure future food security.

Sorghum (*Sorghum bicolor)* and pearl millet (*Pennisetum glaucum***)** are among the most important C4 cereals grown in tropical and subtropical regions [7]. The C4 cereals are staple foods that sustain the population of Sub-Saharan Africa and Asia. They serve as a significant source of energy, essential calories, proteins, and vital micronutrients [8]. Sorghum and pearl millet are among the most drought-resistant cereal crops [9] and thus referred to as climate smart crops. Due to their resilience, these crops offer valuable opportunities for enhanced household food security even under challenging climatic conditions. As a result, smallholder farmers who may lack irrigation facilities and established systems can still produce food for their households and local markets [10,11]. Although these crop species are highly resilient to climate change, low-yield remains a major production challenge [12,13]. Their yields can be significantly reduced due to high-acidity soils, uneven distribution of seasonal rain and poor soil nutrient content [13,14]. A better understanding of plant–microbiome interactions in the rhizosphere soil can unlock new prospects for sustainably enhancing pearl millet and sorghum production in various localities of South Africa.

The rhizosphere serves as a hub for a variety of microorganisms that are highly beneficial to agricultural crop production. These microbial communities are essential for maintaining ecosystem balance, facilitating nutrient cycling, synthesizing hormones that promote crop growth, and enhancing resilience to abiotic stress such as drought, salinity, and extreme temperatures [15]. Furthermore, these microorganisms work in synergy with one another and the crops to improve soil fertility and yield. On the other hand, they are essential indicators to measure soil health [16]. Such microorganisms are crucial for agricultural sustainability and alleviating the effects of climate change.

Understanding the influence, interaction and roles of microbial communities in agricultural environments is essential for promoting sustainable farming practices and its management [17]. Next-generation sequencing such as metagenomics can facilitate a better understanding of microbial communities in the soil and provide genetic information in genomes from both culturable and unculturable organisms [18]. Moreover, the composition and abundance of microbial communities in the soil vary significantly based on soil properties and environmental conditions. In this study, we aimed to use 16S rRNA amplicons to investigate microbial diversity and abundance levels, physicochemical properties and physiological levels of sorghum and pearl millet rhizosphere soil samples from 16 farms in Mpumalanga and Limpopo provinces of South Africa.

## Methods and materials

### Site description and soil sampling

**Fig 1:**
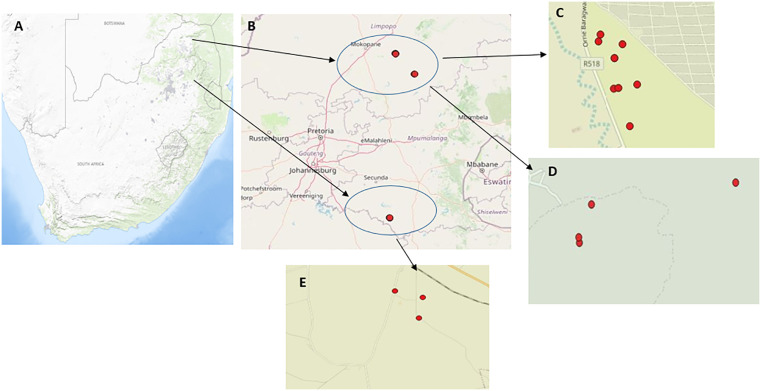
**Map of South Africa showing the two provinces, Mpumalanga and Limpopo, where soil samples were collected (A).**Map indicating the three sampling locations within Limpopo and Mpumalanga provinces **(B)**. Soil sampling sites in Lebowakgomo **(C)**, Maseleseleng **(D)** and Standerton **(E)**.

Rhizosphere soils were collected from sixteen monocroped sorghum and pearl millet farms in Mpumalanga (Standerton) and Limpopo (Jane Furse-Maseleseleng, and Lebowakgomo) provinces, comprising of three pearl millet and thirteen sorghum farms (S1 Table, Fig 1). Sampling was conducted in December 2023. During the sampling period, the average temperature remained at 16–32 °C in Limpopo and 14–26 °C in Mpumalanga, while the average precipitation ranged from 385–800 mm in Limpopo and 570–750 in Mpumalanga [19].

Soil samples were collected from subsistence farms that primarily cultivate sorghum and pearl millet, though they rotate with legumes. The farms used conventional tillage practices to prepare the soil before planting the crops. None of the farms used any fertilizers.

A stratified random sampling approach was used to collect soil samples at a depth of 0–30 cm with a soil auger. The soil samples collected from each farm were then pooled into representative samples as shown in S1 Table. The samples were used to analyze metagenomics, soil physicochemical properties, and community-level physiological profiles.

### Soil physicochemical analysis

For physiochemical analysis, soil samples were sent to the Agricultural Research Council-Soil Climate and Water Analytical Services Division. The physicochemical properties were performed using procedures described in the handbook of standard soil testing methods [20]. Exchangeable cations including calcium (Ca), magnesium (Mg), sodium (Na), and potassium (K) were extracted using 0.1 M ammonium acetate and quantified with an inductively coupled plasma optical emission spectrometer (ICP-OES). Copper (Cu), manganese (Mn), iron (Fe) and zinc (Zn) were extracted with 0.1 M HCl, while aluminium (Al) was extracted in 1.0 KCL and analyzed using ICP-OES. Soil texture (sand, silt, and clay fractions) was determined using the hydrometer method following the Bouyoucos procedure. Available phosphorus (P) was assessed using the Bray 1 extraction method and measured with a flow analyzer. Soil pH was determined in a 1:10 soil-to-water suspension using a calibrated pH meter. Nitrate (NO₃⁻) concentrations were obtained by extraction with 1 M KCl, followed by quantification using a flow analyzer.

### Community-level physiological profiles of rhizosphere soil microbial communities

The microbial community level physiological profiles (CLPP) were analyzed using BIOLOG™ EcoPlates. The plates consist of 96 wells of which 31 wells contain different carbon substrates: 1) carbohydrates: Pyruvic acid Methyl Ester, D-Cellobios, α-D-Lactose, β-Methyl-D-Glucoside, D-Xylose, i-Erythritol, D-Mannitol, N-Acetyl-D-glucoseamine, Glucose-1-phosphate, D,L-αGlycerol phosphate (2) carboxylic acids: D-glucoseaminic acid, D-Galactonic acid γ-Lactone, D-Galacturonic acid, 2-Hydroxy Benzoic acid, 4-Hydroxy Benzoic acid, γ-Amino Butyric acid, Itaconic acid, α-Keto Butyric acid,D-Malic acid, (3) amino acids: L-threonine, L-phenylalanine, glycyl-L-glutamic acid, L-asparagine, L-arginine, L-serine; (4) polymers: α-cyclodextrin, glycogen, tween 80, tween 40; (5) amines/amides: phenylethylamine, putrescine and one well acting as blank control, all in triplicate. Ten grams of the pooled soil samples were suspended in sterile saline, followed by agitating for 20 minutes at 28°C. The samples were serially diluted up to 10 ⁻ ³, and a 120 µl aliquot of the 10 ⁻ ³ soil solution was inoculated into each well of the EcoPlate. The plates were incubated in the dark for 96 hours at 28°C, after which the absorbance was measured at 590 nm using a Chromate Microplate Reader. Microbial response in each microplate was expressed as average well-color development (AWCD) and Shannon–Weaver (*H*’) indices. The AWCD index was determined using the following formula:


$$\begin{array}{c} AWCD\;=\;\Sigma (C-\;R)\;/n\; \end{array}$$

Where c = absorbance of the sample*,* n = number of wells, and R = absorbance in the control well.

The Shannon–Weaver (H’) index was calculated using the following formula:$\;H^{\prime }=-\;\sum_{I=1}^{N}Pi\;(lnPi)$**,**

Where *p*_*i*_ is the ratio of the activity on each substrate to the sum of activities on all substrates.

### DNA extraction and 16S rDNA amplicon sequencing

Genomic DNA was extracted from 34 pooled samples using the ZymoBIOMICS DNA Miniprep kit according to the manufacturer’s protocol. The quality and concentration of genomic DNA were determined using a Qubit™ 4 Fluorometer **(**Thermo Fisher Scientific™). The libraries were prepared following the Illumina 16S Library preparation protocol. Briefly, the V3 and V4 regions of the bacterial 16s rRNA gene were amplified using the KAPA HiFi Hot Start Master Mix PCR kit and universal primers: forward (5’

TCGTCGGCAGCGTCAGATGTGTATAAGAGACAGCCTACGGGNGGCWGCAG) and reverse (5’ GTCTCGTGGGCTCGGAGATGTGTATAAGAGACAGGACTACHVGGGTATCTAATCC) [21]. The size of the PCR product was verified by using a Bioanalyzer DNA 1000 chip; and AMPure XP beads were used to purify the 16S V3 and V4 amplicon. Nextera XT Index kit was used to attach dual indices and Illumina sequencing adapters. A second clean-up of the libraries was performed using AMPure XP beads. The libraries were pooled, denatured and sequenced using the Illumina MiSeq, generating 300 bp paired-end.

### 16S rRNA amplicon sequence data analysis

The raw data for all samples were analyzed in R software (version 4.0.3). The quality of the raw sequencing reads was evaluated using FastQC v0.11.9 [22], and MultiQC v1.12 [23] was utilized to obtain an overview of all the quality control results from FastQC. To improve read quality, Trimmomatic v0.39 [24] was used for the removal of adapter sequences and low-quality bases. Sequenced reads were processed and filtered using the QIIME2 pipeline [25]; DADA2 [26] was utilized for read filtering, error correction, and the identification of Amplicon Sequence Variants (ASVs). To eliminate low-quality bases, the forward reads were truncated to 270 base pairs and reverse reads to 200 base pairs, at the 3’-end, while no trimming was performed at the 5’ end of either forward or reverse reads. Chimera removal was conducted using DADA2’s default settings during the denoising process. The resulting ASV feature table and representative sequences were then exported for downstream analysis using the SILVA v138 classifier [27] with a 99% similarity threshold for bacterial and archaeal species identification.

The phylum-level relative abundance plots were generated directly in phyloseq [28] using the processed ASV feature table and taxonomy table exported from QIIME2. The QIIME2 outputs were imported into R and merged into a single phyloseq object comprising the ASV table, taxonomy table, and sample metadata. Amplicon sequence variants were agglomerated to the phylum level using standard Phyloseq functions (tax_glom, transform_sample_counts) and plots were created using ggplot2 with Phyloseq-formatted output.

Genus level was visualized using a heatmap constructed on a Galaxy server (https://usegalaxy.org/). Quality control of the raw sequence reads was assessed using FastQC (version 0.12.1) [22], followed by trimming with the Trimmomatic program (Version 0.39), [24]. Relative abundance of the 21 most abundant bacterial genera was used to construct a heatmap using heatmap.2 program. Alpha diversity metrics, and beta-diversity metrics were calculated using the Phyloseq and Vegan packages in Rstudio. Alpha diversity was calculated using Observed OTUs, ACE, Chao1, Shannon, Simpson, Inverse Simpson, and Fisher indices to show both species richness and evenness.

Microbial community dissimilarities among samples were calculated using Bray–Curtis distances based on the ASV abundance table in R/RStudio using the vegan package (v2.6-4). Differences in microbial community composition among groups were assessed using permutational multivariate analysis of variance (PERMANOVA) implemented in the adonis2 function with 999 permutations. Crop type (sorghum vs pearl millet) and sampling location were included as explanatory variables to evaluate their relative contributions to microbial community structure.

To identify microbial taxa potentially associated with sorghum and pearl millet rhizospheres, a Random Forest classification analysis was performed using the randomForest package in R/RStudio. The model was trained using the ASV abundance matrix as predictors and crop type as the response variable. A total of 1000 trees were grown, and the number of variables tried at each split was determined automatically by the algorithm. Taxa importance was assessed using the mean decrease in classification accuracy, and the most influential taxa contributing to discrimination between crop types were visualized.

Correlation between environmental variables and community composition was examined using Mantel tests [29], BIO-ENV (bioenv) analysis [30], and distance-based redundancy analysis (dbRDA) [31], implemented via the vegan package [32]. Spearman correlation analysis was conducted using the Hmisc package.

### Statistical analysis on community level physiological profile data

A one-way analysis of variance (ANOVA) was carried out on the CLPP data sets using the general linear model procedure (PROC GLM) in SAS version 9.4 statistical software [33]. The residuals were examined for deviations from normality and outliers causing skewness were removed. Tukey’s Honestly Significant Difference (HSD) Test was used to group means of significant effects (α = 0.05). The data was also subjected to multivariate methods like principal component analysis (PCA) and agglomerative hierarchical clustering using XLSTAT [34] software to visualize and elucidate the relationships between the samples and their attributes. Pearson correlation matrix analysis was conducted using XLSTAT to determine the relationships among soil physicochemical properties, carbon sources, and bacterial phyla [34].

## Results

### Soil physicochemical properties

The results of the soil physicochemical properties among the sixteen farms are shown in S2-S4 Tables. The Mineral levels varied among the farms. Soil samples from the Standerton farms showed higher concentrations of the minerals (Fe, Mn, NO_3_, P, Zn, Ca, and K) compared to the Lebowakgomo and Jane Furse (Maseleseleng farms). Most of the Lebowakgomo farms showed higher levels of Cu, Na, Mg, and CEC, compared to Standerton and Jane Furse farms (S2-S4 Tables). Soils at Standerton farms are sandy clay to sandy clay loam with clay content ranging from 16–36% and dominant sand content ranging from 38–72%. Maseleseleng farms soils are consistently sandy loam, characterized by high sand (74–88%) and low clay (10–18%) and silt contents (2–8%). Lebowakgomo farms soils are predominantly sandy clay to sandy clay loam, with moderate clay (22–40%) and sand (44–68%) proportions. In general, the sand content was higher in all farms. Furthermore, the content of the silt was relatively low in all three localities (S2-S4 Tables). The pH levels in all sixteen farms were comparable, ranging from 5.34 to 6.54.

### Community-level physiological profiles (CLPP) of microorganisms

The metabolic activity of the soil microbial population on the five carbon sources showed significant variation across the farms (Table 1). Higher utilization of carbon substrates in amino acids, amines, carboxylic acids, and polymers was observed in the Jane Furse (Maseleseleng farms), while lower utilization of the carbon source amines was observed in most of the Lebowakgomo farms. The utilization of carbohydrates varied among the farms with Lebo-Mil3B and Stand-Mai3B soil samples showing a higher utilization of carbohydrates. To further analyze the catabolic diversity among the farms, Richness (S), Shannon-Weaver (H), Evenness (E) and AWCD were calculated (Table 1). There were statistically significant differences in the soil AWCD index among the farm sites, p < 0.0001. Stand-Mai3B recorded the highest AWCD while Stand-Sor1A recorded the lowest AWCD. However, most farms from Jane Furse have recorded statistically higher AWCD values. Statistically significant differences in the values of Shannon-Weaver diversity index and Richness were recorded among the farms. The Shannon index ranged from 1.80 to 3.28, with the highest value recorded from the Standerton farm Stand-Mai3B soil sample. The microbial richness ranged from 4.67 to 27, with Stand-Mai 3B having the highest microbial richness and Stand-Sor1A having the lowest microbial richness. There was no difference in the evenness across all farms, except for Math-Sor1C, Stand-Sor1A, Stand-Sor1D, Math-Sor1C and Mase-Sor3E. A dissimilarity dendrogram was constructed according to the Ward method. The dendrogram revealed two major clusters of soil samples, indicating substantial differences in microbial community metabolic activity profiles (S1 Fig). The first cluster (blue) shows that the soils from the farms have similar carbon utilization patterns as compared to the second cluster (red). The second cluster (red) showed distinct carbon utilization patterns among the farms.

**Table 1:** Carbon source utilization by soil microbial communities from 34 soil samples, presented as total AWCD, carboxylic acids, amino acids, polymers, amines, carbohydrates, together with Shannon Weaver index (H), Richness (S) and Evenness (E).

Farm sites	Carbohydrates	Carboxylic Acids	Amino Acids	Polymers	Amines	AWCD	H	S	E
**Lebo-Mill2A**	1.133 ± 0.12^ab^	0.91 ± 0.12^a-f^	0.95 ± 0.05^abc^	0.93 ± 0.05^a-g^	0.89 ± 0.06^a-f^	0.99 ± 0.03^a-d^	3.20 ± 0.04^ab^	24.33 ± 1.15^ab^	1.00 ± 0.01^d^
**Lebo-Mill2B**	0.59 ± 0.09^e-k^	0.29 ± 0.21^j-n^	0.37 ± 0.08^g-k^	0.52 ± 0.22^g-l^	0.47 ± 0.48^d-h^	0.44 ± 0.05^fgh^	2.57 ± 0.18^b-i^	12.67 ± 3.51^efg^	1.03 ± 0.05^d^
**Lebo-Mill3A**	1.01 ± 0.12^a-f^	0.69 ± 0.21^b-i^	0.98 ± 0.20^abc^	0.73 ± 0.21^e-k^	0.86 ± 0.31^a-f^	0.87 ± 0.04^d^	3.04 ± 0.04^a-e^	22.00 ± 1.00^a-d^	0.98 ± 0.01^d^
**Lebo-Mill3B**	1.38 ± 0.11^a^	1.04 ± 0.25^a-d^	1.07 ± 0.09^a^	0.91 ± 0.35^a-h^	0.94 ± 0.15^a-f^	1.13 ± 0.13^abc^	3.20 ± 0.05^ab^	25.67 ± 0.58^a^	0.99 ± 0.01^d^
**Lebo-Mill8**	0.61 ± 0.16^d-j^	0.20 ± 0.17^lmn^	0.32 ± 0.26^h-k^	0.41 ± 0.14^h-l^	0.01 ± 0.01^h^	0.37 ± 0.13^f-i^	2.16 ± 0.48^ij^	9.33 ± 3.06^fgh^	0.98 ± 0.07^d^
**Lebo-Sor1**	0.32 ± 0.02^ijk^	0.43 ± 0.04^g-n^	0.55 ± 0.17^c-j^	0.54 ± 0.03^h-l^	0.55 ± 0.15^c-h^	0.44 ± 0.06^fgh^	2.67 ± 0.13^a-i^	14.33 ± 1.53^ef^	1.01 ± 0.05^d^
**Lebo-Sor4**	0.23 ± 0.08^ijk^	0.14 ± 0.07^mn^	0.34 ± 0.11^h-k^	0.30 ± 0.11^jkl^	0.06 ± 0.104 ^hg^	0.22 ± 0.05^hij^	2.46 ± 0.27^d-i^	8.33 ± 2.08^fgh^	1.18 ± 0.13^bc^
**Lebo-Sor5**	0.54 ± 0.3^g-k^	0.33 ± 0.13^j-n^	0.48 ± 0.10^f-k^	0.60 ± 0.66^g-l^	0.03 ± 0.54^h^	0.44 ± 0.06^fgh^	2.57 ± 0.21^b-i^	12.67 ± 1.15^efg^	1.01 ± 0.05^d^
**Lebo-Sor6**	0.35 ± 0.26^h-k^	0.32 ± 0.11^j-n^	0.41 ± 0.67^g-k^	0.19 ± 0.27^l^	0.18 ± 0.26^f-h^	0.32 ± 0.09^g-j^	2.35 ± 0.28^f-j^	9.33 ± 2.08^fhg^	1.06 ± 0.04^dc^
**Lebo-Sor7**	0.79 ± 0.16^b-h^	0.63 ± 0.14^e-k^	0.51 ± 0.14^d-j^	0.46 ± 0.21^h-l^	0.02 ± 0.30^h^	0.60 ± 0.07^ef^	2.70 ± 0.07^a-i^	14.00 ± 1.73^ef^	1.03 ± 0.03^d^
**Lebo-Sor9**	1.10 ± 0.09^abc^	0.75 ± 0.10^a-h^	0.79 ± 0.20^a-g^	0.71 ± 0.01^f-k^	0.66 ± 0.54^b-h^	0.86 ± 0.06^d^	3.07 ± 0.08^a-d^	21.67 ± 3.01^a-d^	1.00 ± 0.02^d^
**Mase-Sor1A**	0.91 ± 0.01^b-g^	1.06 ± 0.04^abc^	0.96 ± 0.03^abc^	1.30 ± 0.11^ab^	1.24 ± 0.09^a-d^	1.03 ± 0.00^a-d^	3.16 ± 0.01^ba^	23.33 ± 1.15^abc^	1.00 ± 0.01^d^
**Mase-Sor2A**	1.22 ± 0.04^ab^	0.87 ± 0.07^a-f^	1.11 ± 0.04^a^	0.77 ± 0.01^c-k^	1.46 ± 0.18^a^	1.06 ± 0.03^a-d^	3.20 ± 0.03^ba^	25.00 ± 1.73^ba^	1.00 ± 0.01^d^
**Mase-Sor2B**	0.82 ± 0.05^b-g^	0.82 ± 0.06^a-g^	0.73 ± 0.11^a-h^	0.89 ± 0.05^e-j^	1.07 ± 0.16^a-f^	0.83 ± 0.06^de^	3.13 ± 0.05^a-d^	23.67 ± 1.53^ba^	0.99 ± 0.00^d^
**Mase-Sor2C**	0.89 ± 0.01^b-g^	0.67 ± 0.07^c-j^	0.94 ± 0.06^a-d^	1.40 ± 0.05^a^	1.17 ± 0.08^a-d^	0.92 ± 0.02^dc^	3.17 ± 0.02^ba^	23.00 ± 0.00^abc^	1.01 ± 0.00^d^
**Mase-Sor2D**	0.97 ± 0.03^a-g^	0.68 ± 0.02^c-j^	1.00 ± 0.06^ab^	1.25 ± 0.05^a-d^	0.94 ± 0.02^a-f^	0.93 ± 0.04^dc^	3.07 ± 0.01^a-d^	21.67 ± 1.15^a-d^	1.00 ± 0.02^d^
**Mase-Sor2E**	0.58 ± 0.07^f-k^	0.74 ± 0.05^a-i^	1.02 ± 0.04^a^	1.23 ± 0.01^a-e^	1.33 ± 0.13^a-c^	0.84 ± 0.03^d^	3.00 ± 0.03^a-g^	21.00 ± 0.00^a-d^	0.99 ± 0.01^d^
**Mase-Sor2F**	1.04 ± 0.03^a-e^	1.05 ± 0.05^abc^	1.07 ± 0.07^a^	0.35 ± 0.12^kjl^	1.26 ± 0.05^a-c^	0.97 ± 0.03^a-d^	3.16 ± 0.01^ba^	25.00 ± 0.00^ba^	0.98 ± 0.00^d^
**Mase-Sor3A**	0.96 ± 0.10^a-g^	0.87 ± 0.02^a-f^	0.99 ± 0.03^ab^	1.29 ± 0.03^ab^	1.28 ± 0.07^a-c^	1.00 ± 0.04^a-d^	3.16 ± 0.05^ba^	23.00 ± 1.00^bac^	1.01 ± 0.01^d^
**Mase-Sor3B**	1.06 ± 0.08^a-d^	0.82 ± 0.06^a-g^	1.06 ± 0.05^a^	1.26 ± 0.02^abc^	1.26 ± 0.04^a-c^	1.03 ± 0.04^a-d^	3.17 ± 0.04^ba^	23.67 ± 0.58^ba^	1.00 ± 0.01^d^
**Mase-Sor3C**	1.17 ± 0.13^ab^	1.11 ± 0.07^a^	1.12 ± 0.03^a^	1.32 ± 0.09b^a^	1.47 ± 0.04^a^	1.18 ± 0.04^a-d^	3.23 ± 0.02^ba^	25.33 ± 1.53^ba^	1.00 ± 0.01^d^
**Mase-Sor3D**	0.98 ± 0.11^a-g^	1.02 ± 0.01^a-d^	0.89 ± 0.14^a-f^	0.90 ± 0.11^a-i^	1.32 ± 0.11^a-c^	0.99 ± 0.05^a-d^	3.22 ± 0.02^ba^	25.67 ± 1.53^a^	0.99 ± 0.01^d^
**Mase-Sor3E**	0.28 ± 0.19^ijk^	0.35 ± 0.25^h-n^	0.38 ± 0.17^g-k^	0.53 ± 0.08^g-l^	0.25 ± 0.30^f-h^	0.35 ± 0.12^ghi^	2.48 ± 0.41^d-i^	9.33 ± 3.06^fhg^	1.13 ± 0.01^dc^
**Math-Sor1A**	0.93 ± 0.08^a-g^	0.82 ± 0.00^a-g^	0.92 ± 0.02^a-e^	1.17 ± 0.06^a-f^	1.23 ± 0.04^a-d^	0.95 ± 0.03^bcd^	3.19 ± 0.00^ba^	24.00 ± 1.00^ba^	1.00 ± 0.01^d^
**Math-Sor1B**	0.24 ± 0.13^ikj^	0.09 ± 0.04^n^	0.21 ± 0.24^ijk^	0.30 ± 0.07^jkl^	0.04 ± 0.07^h^	0.18 ± 0.05^ij^	2.38 ± 0.18^f-j^	6.33 ± 1.53 ^hg^	1.31 ± 0.1^ba^
**Math-Sor1C**	0.15 ± 0.08^k^	0.13 ± 0.11^nm^	0.13 ± 0.11^k^	0.26 ± 0.21 ^kl^	0.02 ± 0.03^h^	0.15 ± 0.05^ij^	1.80 ± 0.27^j^	5.00 ± 1.00^h^	1.13 ± 0.057^dc^
**Stand-Mai3A**	1.10 ± 0.20^abc^	0.77 ± 0.25^a-g^	1.11 ± 0.16^a^	0.94 ± 0.22^a-g^	0.76 ± 0.48^a-h^	0.97 ± 0.1^a-d^	3.07 ± 0.07^a-d^	22.00 ± 1.73^a-d^	0.99 ± 0.01^d^
**Stand-Mai3B**	1.38 ± 0.07^a^	1.09 ± 0.02^ab^	1.10 ± 0.03^a^	1.13 ± 0.08^a-f^	1.35 ± 0.19^ab^	1.21 ± 0.02^a^	3.28 ± 0.01^a^	27.00 ± 0.00^a^	0.99 ± 0.00^d^
**Stand-Sor1A**	0.15 ± 0.14^jk^	0.05 ± 0.04^n^	0.08 ± 0.08^k^	0.15 ± 0.07^l^	0.05 ± 0.01^gh^	0.10 ± 0.08^j^	2.33 ± 0.55^g-j^	4.67 ± 3.79^h^	1.39 ± 0.22^a^
**Stand-Sor1B**	0.21 ± 0.24^ijk^	0.24 ± 0.14^k-n^	0.46 ± 0.24^f-k^	0.40 ± 0.19^i-l^	0.25 ± 0.36^fgh^	0.29 ± 0.16^g-j^	2.29 ± 0.56^hij^	8.67 ± 4.16^fgh^	1.13 ± 0.07^dc^
**Stand-Sor1C**	0.57 ± 0.22^f-k^	0.56 ± 0.27^e-l^	0.32 ± 0.1^h-k^	0.47 ± 0.37^g-l^	0.70 ± 0.06^a-h^	0.51 ± 0.17^fg^	2.87 ± 0.13^a-h^	17.00 ± 3.60^cde^	1.02 ± 0.04^d^
**Stand-Sor1D**	0.66 ± 0.18^c-i^	0.44 ± 0.09^g-n^	0.51 ± 0.13^e-j^	0.46 ± 0.17^g-l^	0.20 ± 0.20^hgf^	0.51 ± 0.05^fg^	2.89 ± 0.05^a-h^	16.33 ± 0.58^de^	1.03 ± 0.01^dc^
**Stand-Sor2A**	0.59 ± 0.16^f-h^	0.53 ± 0.12^f-m^	0.58 ± 0.21^b-i^	0.27 ± 0.07 ^kl^	0.36 ± 0.31^e-h^	0.51 ± 0.09^fg^	3.01 ± 0.15^a-f^	19.00 ± 2.65^b-e^	1.03 ± 0.01^d^
**Stand-Sor2B**	1.10 ± 0.20^ab^	0.96 ± 0.10^a-e^	1.05 ± 0.17^a^	0.75 ± 0.11^d-k^	0.83 ± 0.70^a-g^	0.99 ± 0.07^a-d^	3.13 ± 0.02^abc^	24.67 ± 0.58^ab^	0.98 ± 0.01^d^
**F-statistic**	20.85^**^	19.62^**^	19.47^**^	18.49^**^	13.64^**^	62.36^**^	10.61**	38.87^**^	9.91^**^

**Keynotes:** Values (mean ± SD) with different letters in a column are significantly different. **indicates significant difference at p *<* 0.0001. (Stand-Standerton farms, Lebo-Lebowakgomo, Mase and Math farms- Jane Furse). Carbon-source utilization values represent optical density measured at 590 nm (OD₅₉_0_)

### Microbial distribution across the soil samples at phylum level

**Fig 2:**
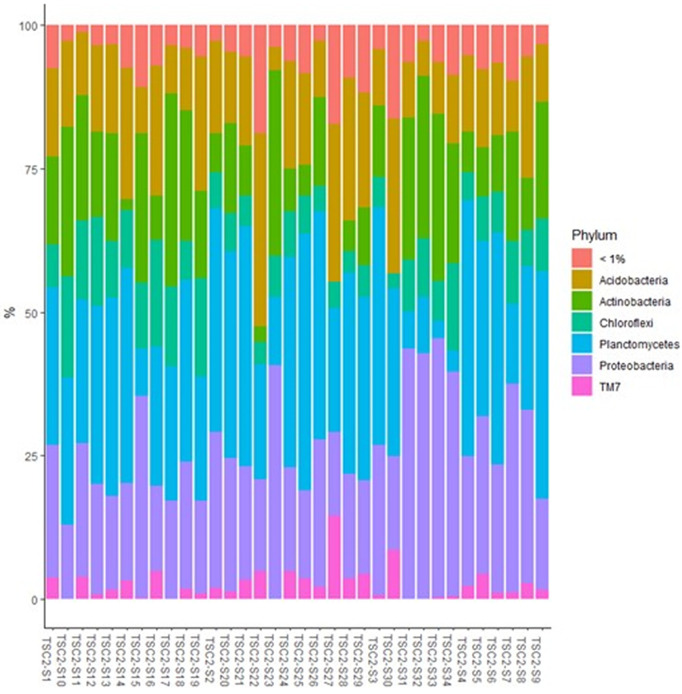
Relative abundance of bacterial phyla across 34 soil samples.

**Fig 3:**
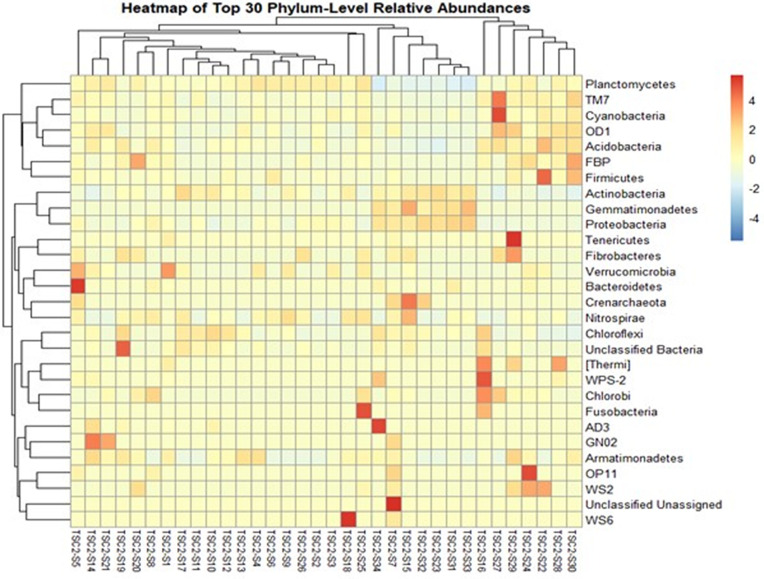
Heatmap illustrating the relative abundance of top 30 bacterial phyla across 34 soil samples. **Keynotes**: TSC2-S1-Stand-Sor1A, TSC2-S2-Stand-Sor1B, TSC2-S3-Stand-Sor1C, TSC2-S4-Stand-Sor1D, TSC2-S5-Stand-Sor2A, TSC2-S6-Stand-Sor2B, TSC2-S7-Stand-Mai3A, TSC2-S8-Stand-Mai3B, TSC2-S9-Mase-Sor1A, TSC2-S10-Mase-Sor2A, TSC2-S11-Mase-Sor2B, TSC2-S12-Mase-Sor2C, TSC2-S13-Mase-Sor2D, TSC2-S14-Mase-Sor2E, TSC2-S15-Mase-Sor2F, TSC2-S16-Mase-Sor3A, TSC2-S17-Mase-Sor3B, TSC2-S18-Mase-Sor3C, TSC2-S19-Mase-Sor3D, TSC2-S20-Math-Sor1A, TSC2-S21-Math-Sor1B, TSC2-S22-Math-Sor1C, TSC2-S23-Lebo-Sor1, TSC2-S24-Lebo-Mill2A, TSC2-S25-Lebo-Mill2B, TSC2-S26-Lebo-Mill3A, TSC2-S27-Lebo-Mill3B, TSC2-S28-Lebo-Sor4, TSC2-S29-Lebo-Sor5, TSC2-S30-Lebo-Sor6, TSC2-S31-Lebo-Sor7, TSC2-S32-Lebo-Mill8, TSC2-S33-Lebo-Sor9, TSC2-S34-Mase-Sor3E.

The bacterial community among the farms varies, Planctomycetes and Proteobacteria were dominant in most samples, while other phyla like Chloroflexi, Actinobacteria and TM7 were present in lower but variable proportions (Fig 2). Groups <1% represent low-abundance phyla or taxa that collectively make up less than 1% of the community in each sample. The heatmap showed that the most abundant phylum in some of the farms includes Planctomycetes*,* Proteobacteria, and Actinobacteria (Fig 3). Planctomycetes phyla was more observed in samples Stand-Sor1B, Stand-Sor1C, Stand-Sor1D, Stand-Sor2B, Mase-Sor1A, Math-Sor1B, Lebo-Mill2B and Lebo-Mill3A. Proteobacteria, Actinobacteria and Gemmatimonodetes were predominant in samples Mase-Sor3B, Lebo-Mill3B, Lebo-Sor7, Lebo-Mill8, Lebo-Sor9 and Mase-Sor3E.

### Microbial distribution across the soil samples at genus level

**Fig 4:**
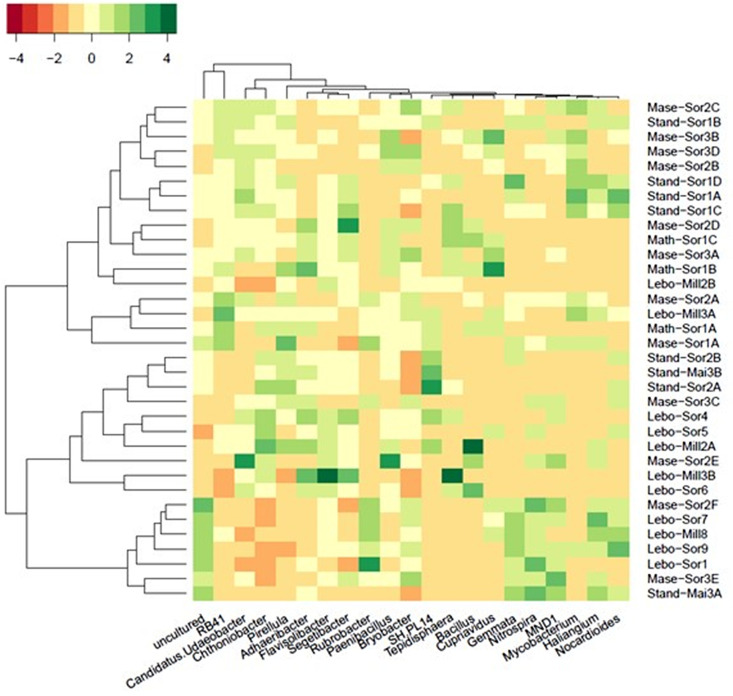
Heatmap illustrating the relative abundances of the top 21 bacterial genera across 34 soil samples.

The most predominant genera at the genus level were *RB41, Chthoniobacter, Candidatus Udaeobacter* and *Pirellula* in most farms (Fig 4)*.* The *RB41* genus was identified in all farms except for one farm site (Lebo-Mill3B), with higher abundance observed in Lebo-Mill3A (35.39%) and Mase-Sor2A (30.26%). The genus *Chthoniobacter* showed a higher abundance in soil samples from Lebo-Mill2A (18.23%), Lebo-Sor5 (16.80%), Lebo-Sor4 (14.98%) and Stand-Sor2A (13.77%) farm sites. *Candidatus Udaeobacter* was more predominant in sample Mase-Sor2E (20.41%) while *Pirellula* was more predominant in sample Mase-Sor1A (14.69%)*. Mycobacterium, Haliangium,* and *Nocardioides* genus were observed in low abundance levels in all the farms*.*

### Microbial distribution across the location at phylum level

**Fig 5:**
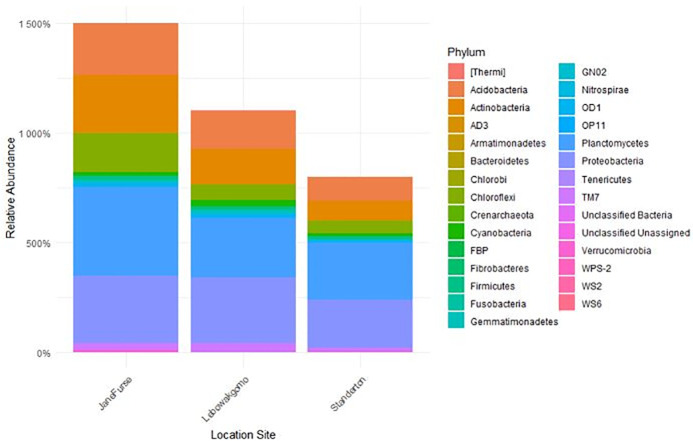
Microbial phylum-level distribution across the locations (Jane Furse, Lebowakgomo, and Standerton).

The soil samples were analysed at the phylum level based on their collection sites in Limpopo (Jane Furse, Lebowakgomo) and Mpumalanga (Standerton). Jane Furse recorded the highest total microbial abundance compared to Lebowakgomo and Standerton (Fig 5). Planctomycetes and Proteobacteria were more predominant in Jane Furse, while Actinobacteria, Acidobacteria and Chlorofexi also contributed significantly. In Lebowakgomo, microbial abundance were slightly lower than in Jane Furse. Moreover, Standerton soils exhibited the lowest total microbial abundance, although it shared the same dominant phyla as Jane Furse and Lebowakgomo. *Acidobacteria* was more dominant in Jane Furse and Lebowakgomo. Proteobacteria remained consistent across all sites but is relatively less dominant in Standerton. Variations in relative abundances indicate that environmental or geographical factors may influence the microbial ecosystems at each site.

### Microbial distribution across soil samples from the two provinces

The microbial community in Limpopo has a significantly higher relative abundance compared to Mpumalanga. Planctomycetes and Proteobacteria were found to be the most dominant in both provinces. Other phyla, such as Acidobacteria, Actinobacteria and Chlorofexi also contributed noticeably to both provinces (S2 Fig). Though both provinces shared many of the same phyla, the relative abundance of each phylum varied significantly between the two regions.

### Alpha diversity by province and location

**Fig 6:**
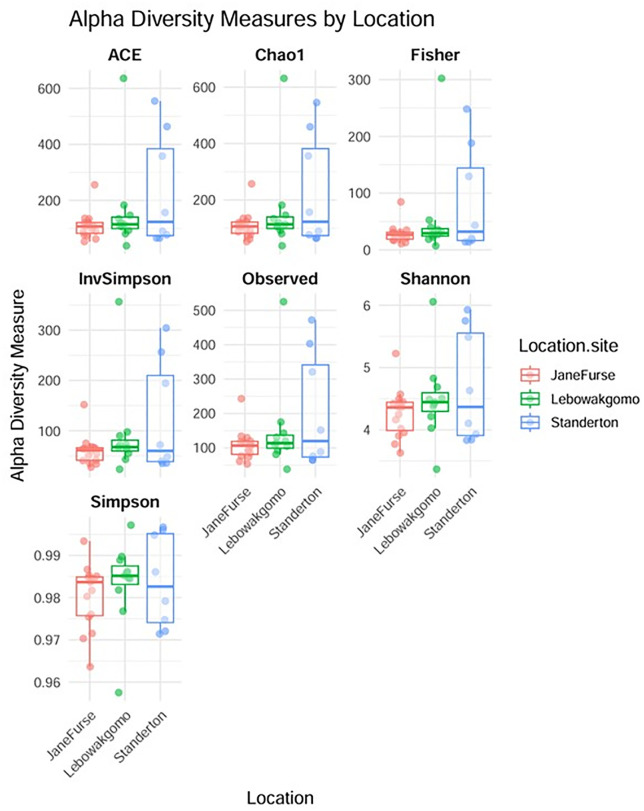
Alpha diversity measures between locations (Jane Furse, Lebowakgomo and Standerton).

Alpha diversity was analyzed in terms of observed OTUs, ACE, Chao1, Shannon, Simpson, InvSimpson and Fisher to reflect the diversity and abundance of microbial communities within the three locations (Jane Furse, Lebowakgomo and Standerton) (Fig 6). Jane Furse consistently showed lower diversity measures, and Lebowakgomo’s diversity measures were moderate and showed less variation compared to Standerton. Species richness (ACE, Chao1) of Lebowakgomo is similar to Jane Furse, but diversity metrics like Shannon and Inverse Simpson were slightly higher. Kruskal–Wallis tests indicated no statistically significant differences in alpha diversity measures between the three sites (p > 0.34). This suggests that, while descriptive patterns are visible in the boxplots, these differences are not statistically supported. S3 Fig illustrates the alpha diversity metrics for the provinces, Limpopo and Mpumalanga. Across all measures, Mpumalanga consistently exhibited greater microbial diversity than Limpopo. This may indicate a more heterogeneous environment in Mpumalanga influenced by environmental factors that promote higher species richness and evenness.

### Beta diversity by location sites

**Fig 7:**
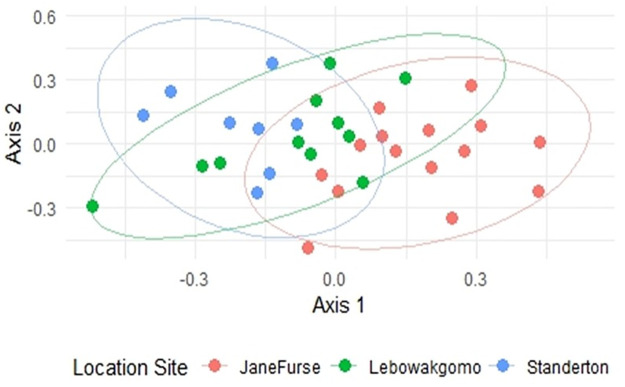
NMDS ordination of microbial communities by location site.

Beta diversity analysis was used to explore the dissimilarity among microbial communities from soil samples collected from Jane Furse, Lebowakgomo and Standerton. The non-multidimensional scaling (NMDS) analysis revealed that samples from the same location clustered together, indicating that microbial community composition is more similar within locations (Fig 7). The overlap between the ellipses for Lebowakgomo and the other two sites suggests shared microbial communities or higher variability in this location. Standerton showed greater dispersion, indicating higher variability in microbial communities. Jane Furse and Lebowakgomo showed less spread, indicating more consistent microbial composition.

The PERMANOVA (adonis2) analysis was used to test the influence of province and location site on microbial community composition, using Bray-Curtis distances to measure dissimilarity. The PERMANOVA results indicate that both province and location significantly influence microbial community composition, with location explaining a larger portion of variance (7.38%) compared to province (3.68%). Both results were statistically significant, with p-values of 0.003 and 0.001, respectively**.** To determine whether host crop influenced rhizosphere microbial communities, A PERMANOVA analysis based on Bray–Curtis dissimilarity was performed. The analysis showed that crop type did not significantly influence rhizosphere bacterial community composition (R² = 0.031, p = 0.286) (S4 Fig). Random Forest analysis identified several taxa contributing to discrimination between sorghum and pearl millet rhizosphere samples, including Chloroflexi, Balneimonas, Ellin6075, TM7, and Solibacterales. However, the importance values were generally low, indicating limited discriminatory power between the two crop rhizospheres (S5 Fig).

**Fig 8:**
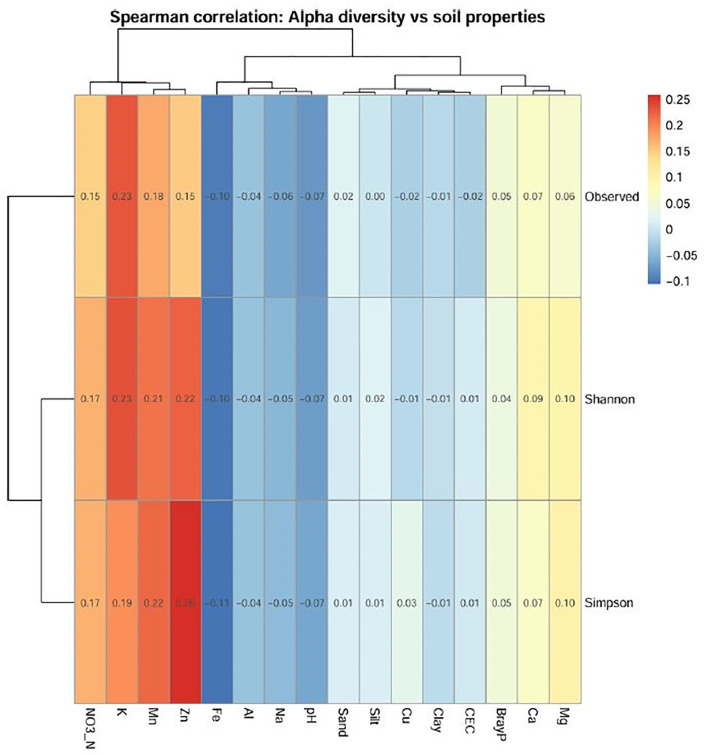
A Spearman correlation analysis of soil physicochemical properties and microbial diversity at phyla level.

All alpha diversity metrics were calculated on rarefied ASV tables to ensure equal sequencing depth across samples. No significant differences were detected among the three sites (Kruskal–Wallis, p > 0.34). To assess the influence of soil physicochemical properties within-sample variation in diversity, Spearman rank correlations were performed between alpha diversity indices and soil variables. Only weak associations were observed, and none remained significant after multiple-test correction, indicating that soil chemistry did not strongly influence within-sample microbial diversity (Fig 8).

**Fig 9:**
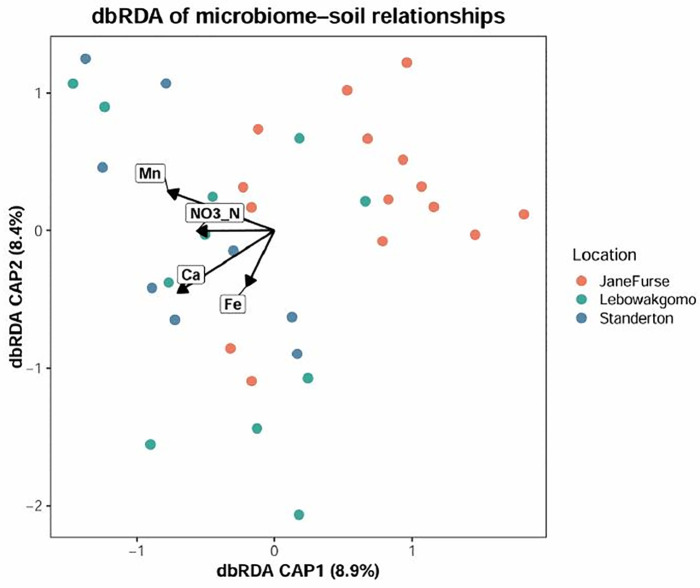
Distance-based redundancy analysis showing correlation between physicochemical properties and microbial communities of the 34 samples across the three localities.

However, the beta diversity analyses revealed clear relationships between physiochemical properties and community composition. The Mantel test demonstrated a significant correlation between community dissimilarity and overall soil physicochemistry (r = 0.228, p = 0.0057), demonstrating that multivariate changes in soil properties are linked to changes in bacterial community composition. BIOENV analysis identified a subset of variables (Cu, Fe, Mn, Silt, Zn, Ca) that best explained Bray–Curtis dissimilarities (p = 0.34). Using dbRDA, soil physicochemical variables collectively explained 46.8% of the total variation in community composition, indicating a substantial environmental effect. Among all the measured factors, Mn, Fe, NO₃-N and Ca were significant drivers of beta diversity (p < 0.05) (Fig 9). Other factors including pH, K, Na, Cu, Mg, Zn and soil texture were not significantly correlated with the bacteria community composition.

### Correlation between bacterial phyla, soil physicochemical properties and carbon sources

The correlation analysis among the carbon sources revealed several significant relationships (P < 0.05, P < 0.001) (S1 File). Carbohydrates showed a significant negative correlation with Na, while carboxylic acids, amino acids, amines, and polymers were negatively with Mg and Na (p < 0.05). Polymers and amines negatively correlated with CEC (p < 0.001). All carbon sources, excluding carbohydrates, showed a strongly significant positive correlation with sand soil texture and a highly significant negative correlation with clay and silt (P < 0.05).

The correlation analysis revealed various significant relationships between bacterial phyla and soil physicochemical properties as well as carbon sources (p < 0.05) (S1 File). Acidobacteria showed a negative correlation with Na, while Proteobacteria showed a positive correlation with Na. Actinobacteria were negatively correlated with Mn and K, while Chlorobi showed a positive correlation with K. Armatimonadetes and WS6 negatively correlated with soil pH. Chlorobi positively correlated with K, while Gemmatimonadetes negatively correlated with K. OP11 was negatively correlated with pH but positively correlated with K. Bacteroidetes showed positive correlations with Al and NO₃-N. TM7 showed positive correlations with Cu, Mn, K, and CEC while Cyanobacteria were positively correlated with Cu, Mn, and CEC. In addition, unclassified bacterial groups were positively correlated with K but negatively correlated with Ca, Mg, and CEC.

Firmicutes showed negative correlations with carbohydrates, carboxylic acids, amino acids, and amines. In contrast, Nitrospirae were positively correlated with carbohydrates, carboxylic acids, amino acids, and amines, indicating opposite responses to these substrates. Similarly, OD1 showed negative correlations with carboxylic acids and amines. Soil texture also influenced certain phyla. Chloroflexi showed negative correlations with Mn, Ca, Mg, and CEC, but positive correlations with carboxylic acids, amino acids, polymers, and amines. Chloroflexi was also influenced by soil texture, the phyla negatively correlated with clay and silt and strongly correlated with sand.

## Discussion

Microorganisms play a crucial role in the agricultural ecosystem and productivity. They perform various functions such as regulating soil properties and fertility, nutrient cycling, inhibiting pathogens, enhancing plant growth and resistance to abiotic stresses, such as drought and heavy metal pollution [35,36]. In this study, the soil’s physicochemical properties, microbial community level, and 16S rRNA metagenomics provided insight into the health and microbial diversity of the soil among farms in Standerton, Jane Furse, and Lebowakgomo.

The soil physicochemical properties varied among the farms, with pH ranging from 5.3 to 6.4. While the metabolic activity of the soil microbial population on the five carbon sources showed significant variation across the farms, with some farms showing higher microbial richness compared to others. Previous studies reported that acidic and alkaline soils usually exhibit lower microbial richness compared to soils with slightly acidic to neutral pH [37,38]. Our study revealed that farms with slightly acidic soil pH (5.67–6.0), including Stand-Mai3B, Mase-Sor2A, Mase-Sor3D, Lebo-Mill3B, Math-Sor1A, and Stand-Sor2B, exhibited the highest microbial richness. However, some farms within this pH range (Stand-Sor1A, Stand-Sor1B, Math-Sor1B and Math-Sor1C) showed lower microbial richness levels. This variation may be influenced by other physicochemical factors such as total soil organic carbon, nitrogen, phosphorus, and soil type [39]. The soil microbial communities utilized various carbon substrates at different rates. This variation may be due to differences in their structural composition and associated functional guilds within each community. However, carbohydrates were the most utilized carbon source within the samples. Similar results on the utilization of carbohydrates by soil microorganisms were reported by other studies [40–42]. In addition, Jane Furse (Maseleseleng) soils showed higher utilization of amino acids, amines, carboxylic acids, and polymers. This suggests that the microbial communities in these soils are actively involved in nutrient cycling and the decomposition of organic matter through processes such as protein degradation, deamination, and the breakdown of complex organic compounds [43,44]. This functional activity aligns with the higher AWCD values and higher microbial richness observed in most Jane Furse farms, suggesting that these soils hosts a diverse community of microorganisms which can utilize a wide range of carbon sources. A rich microbial community can improve soil functions such as water retention, organic matter decomposition and nutrient cycling, making the soil more resilient to environmental stresses like drought and diseases [45].

### Microbial abundance and diversity

It was observed that the rhizosphere soils of all sorghum and pearl millet farms were dominated by the same microbial phylum. The most dominant phyla in the farms were Planctomycetes and Proteobacteria, Actinobacteria and Acidobacteria, which is in agreement with previous studies. A study by Kumar et al. [46] reported Proteobacteria as the most predominant phyla in sorghum rhizosphere soil. Proteobacteria include a wide variety of bacterial groups with versatile metabolic capabilities, enabling them to survive and adapt in various environmental conditions [46]. A previous study reporting on different breed lines of pearl millet rhizosphere diversity found Actinobacteria and Proteobacteria to be the most dominant phyla [13]. Planctomycetes was found to be one of the dominant phyla in the maize rhizosphere [47].

Proteobacteria have been reported to play a key role in numerous processes, including nitrogen, carbon and sulfur cycling, which are essential for nutrient availability and uptake in plants. Based on known literature, Acitinobacteria are potential bioinoculants/biofertilizers due to their ability to solubilize phosphorus, zinc, and potassium, as well as their production of iron-chelating agents and plant-growth-promoting hormones such as indole acetic acid [45,47]. In addition, Planctomycetes are reported to have potential in contributing significantly to soil nutrient degradation, such as nitrogen, potassium and phosphorus, and are involved in facilitating anaerobic ammonium oxidation [48]. At the genus level, *RB41,* belonging to the phylum Acidobacteria, was the predominant genus across all soil samples, although its relative abundance varied (Fig 4). The RB41 genus is recognized as a rhizosphere-associated bacterium; which, based on literature is known to play a significant role in maintaining soil metabolic stability and biogeochemical cycling under hostile environmental conditions [49]. These microorganisms have a potential of utilizing carbon sources and nutrients in the soil and participate in the soil carbon cycle [50].

The alpha diversity analysis across the three locations (Jane Furse, Lebowakgomo, and Standerton) revealed no significant differences in microbial richness or diversity. Although Standerton displayed slightly higher values, the large variance within samples resulted in non-significant statistical outcomes (P > 0.34). This indicates that microbial communities within the three locations share similar diversity and the observed differences may reflect normal environmental variability. Additionally, the amount of organic matter, and its composition may influence both the microbial abundance and their diversity [51,52]. The spearman correlation analysis further supported these results, showing that the number of bacterial richness and evenness were similar across all three study sites. Diversity within samples was not driven by the soil physicochemical properties. Previous studies have shown that when soil samples have similar environmental properties, bacterial richness and evenness frequently show limited variation in bacterial diversity [53].

The PERMANOVA results demonstrated that both province and location significantly influenced microbial community composition, with location explaining a greater proportion of the variance (7.38%) than province (3.68%). Despite the significant effects of province and location, a substantial proportion of the variance remained unexplained, suggesting that additional environmental and agronomic variables may contribute to microbial community dynamics. These may include factors such as soil moisture at the time of sampling, crop rotation practices, and tillage intensity [54,55].

The PERMANOVA analysis showed that sorghum vs. pearl millet did not significantly influence rhizosphere bacterial community composition. Sorghum and pearl millet are both C4 grasses belonging to the Poaceae family and they share similar root architectures and patterns of rhizodeposition [56–58]. Root exudates are known to play an important role in shaping rhizosphere microbial communities by providing carbon sources and signalling compounds that influence microbial recruitment [59]. Due to these physiological similarities, the two crops may recruit comparable rhizosphere microbial assemblages, resulting in the limited host-associated differences observed in this study. In contrast**,** site-associated environmental factors appeared to exert a stronger influence on microbial community composition, as demonstrated by the significant PERMANOVA effect of location. This suggests that soil physicochemical conditions and local environmental factors may play a more dominant role than host identity in structuring rhizosphere bacterial communities in these agricultural systems**.**

Random Forest analysis identified several taxa contributing to discrimination between sorghum and pearl millet rhizosphere samples, including members of Chloroflexi, Balneimonas, Ellin6075, TM7, and Solibacterales. However, the importance values were generally low, indicating limited discriminatory power between the two crop rhizospheres**,** which is consistent with the PERMANOVA results showing no significant crop-type effect on overall microbial community composition.

The dbRDA indicated that Mn, Fe, NO₃-N and Ca were the key factors driving the variations in the bacterial community structure which is similar to previous studies [60,61]. The observed variation in Mn, Fe, NO₃-N, and Ca concentrations may result from both crop rotation and inherent soil properties such as soil texture, parent material and mineral composition. Sorghum and Pearl millet is reported to be rich in iron [62] therefore, the release of iron-enriched root exudates may contribute to the elevated soil iron concentrations observed in this study. The farms rotate the C4 crops with legumes. The rotation of legumes may contribute to the increased NO₃-N levels through biological nitrogen fixation process which explains the elevated NO₃-N levels observed in the soils. Furthermore, decomposition of the rotational legume residues can increase nitrate availability in the soil [63,64]. In addition, incorporating crop residues through conventional tillage can facilitate the gradual decomposition of organic matter, which mobilizes Fe and enhances its availability in the soil [65].

Soil pH is considered a key driver of soil bacterial community structure. However, in this study, pH was not significantly correlated with the bacterial community composition, which is consistent with previous studies [66]. Similarly, Zhang et al. [67] reported that soil bacterial community composition is altered more by soil nutrient availability than pH.

### Correlation analysis of bacterial phyla, soil physicochemical properties and carbon sources

The correlation analysis revealed that several bacterial phyla were strongly associated with soil physicochemical properties and available carbon sources, highlighting the role of nutrient availability and soil chemistry in shaping microbial community structure [68, 69]. Acidobacteria negatively correlated with Na, whereas proteobacteria showed a positive correlation which is consistent with other studies [70–72]. Chloroflexi showed a negative correlation with clay and silt but a positive correlation with sand, contrasting with other studies that reported a positive correlation with clay and silt [73].

Most phyla were positively correlated with specific carbon sources while other phyla, such as Firmicutes and OD1 revealed a negative correlation. This further emphasize that carbon availability significantly influences the bacterial community composition in agricultural soils [68]. Similarly, previous studies have shown that carbon sources such as carbohydrates, amino acids, and carboxylic acids are key drivers of microbial metabolic activity and community structure [74].

## Conclusion

This study investigated the bacterial microbiome diversity and abundance in 34 soil samples pooled from sixteen farms in Limpopo and Mpumalanga. Overall, results revealed that physicochemical properties play a key role in shaping the microbial community systems in the soil. A variation in microbial richness was observed throughout the farms. The 16S rRNA amplicon sequencing results revealed that the most predominant phylum in all localities are Planctomycetes, Proteobacteria, Actinobacteria, Acidobacteria and Chlorofexi, which are known to improve soil fertility and crop production. Carbon utilization, soil physicochemical properties and soil microbial community structure are crucial indicators of a healthy soil. Our study reveals new insight into microbial carbon utilization, physicochemical properties, microbial richness, and diversity from sorghum and pearl millet farms in Limpopo and Mpumalanga provinces, which might help improve the sustainability of agriculture in these and similar areas.

## Supporting information

S1 File Pearson correlation matrix relationship among soil physicochemical, carbon sources and bacterial phyla. (XLSX)

S1 Fig Dendrogram illustrating the dissimilarity of the carbon utilization patterns between 34 soil sample. (DOCX)

S2 Fig Comparison of microbial phylum-Level distribution between Province (Limpopo and Mpumalanga). (TIFF)

S3 Fig Alpha diversity measures between Limpopo and Mpumalanga. (TIFF)

S4 Fig Non-metric multidimensional scaling (NMDS) ordination of rhizosphere bacterial communities based on Bray– Curtis dissimilarity, colored by crop type. (TIFF)

S5 Fig Random Forest importance plot showing microbial taxa contributing to discrimination between sorghum and pearl millet rhizosphere samples. Importance values represent the mean decrease in classification accuracy when each taxon is removed from the model.
(TIFF)

S1 Table Farm sites where soil samples were collected in the two provinces. (DOCX)

S2 Table Physical and chemical properties of soil samples from Standerton farms. (DOCX)

S3 Table Physical and chemical properties of soil samples from Jane Furse farms. (DOCX)

S4 Table Physical and chemical properties of soil samples from Lebowakgomo farms. (DOCX)
