# Supplementary material for: 16S rRNA-based metagenomics insights into the microbial diversity and functional attributes of soils from the rhizosphere of selected C4 crops of farms in Mpumalanga and Limpopo provinces, South Africa
Source: PLoS One. 2026 Jun 15;21(6):e0347776. doi: 10.1371/journal.pone.0347776 (PMC13268165; doi:10.1371/journal.pone.0347776)
Supplement: S1 Fig — (DOCX) [file pone.0347776.s002.docx]

**S1 Fig. Dendrogram illustrating the dissimilarity of the carbon utilization patterns between 34 soil samples.**
