# Supplementary figures and images for: 16S rRNA-based metagenomics insights into the microbial diversity and functional attributes of soils from the rhizosphere of selected C4 crops of farms in Mpumalanga and Limpopo provinces, South Africa
Source: PLoS One. 2026 Jun 15;21(6):e0347776. doi: 10.1371/journal.pone.0347776 (PMC13268165; doi:10.1371/journal.pone.0347776)

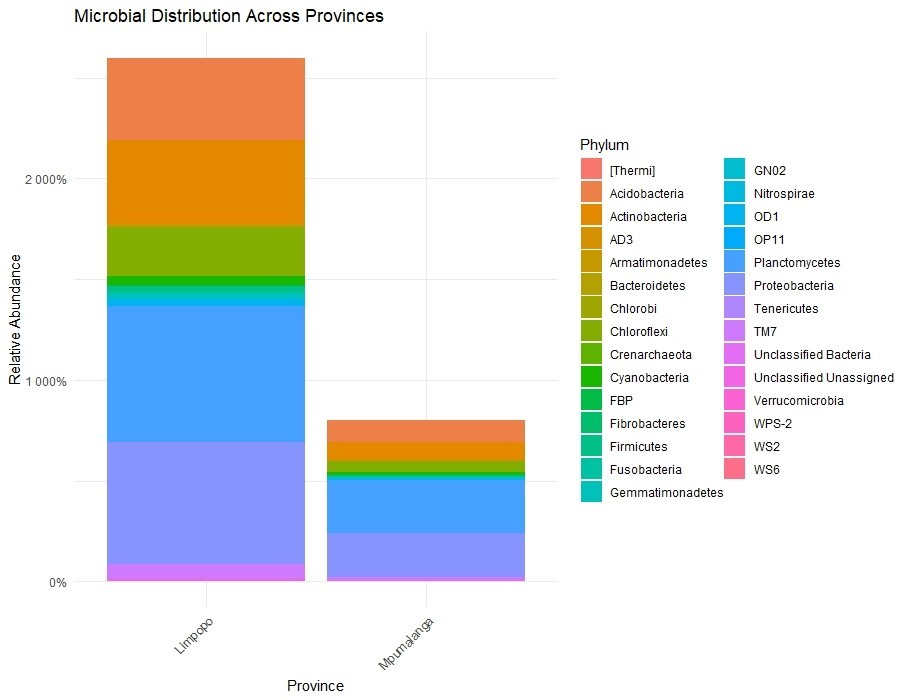

Supplement: S2 Fig — (TIFF) [file pone.0347776.s003.tiff]

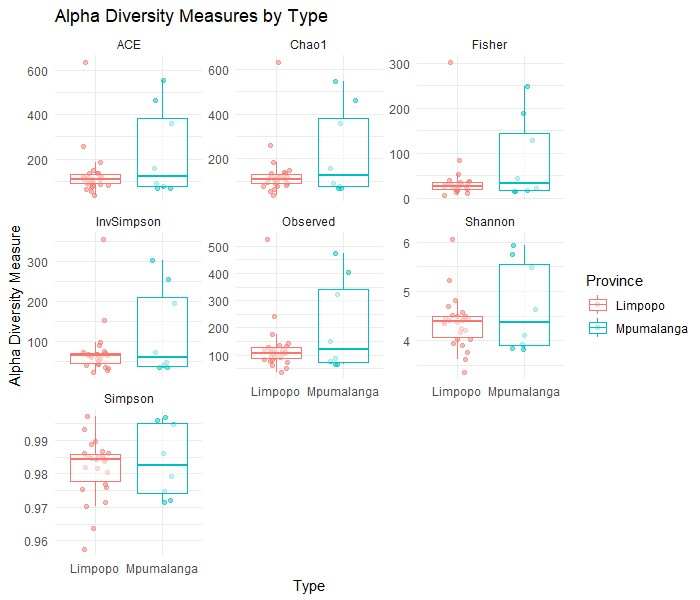

Supplement: S3 Fig — (TIFF) [file pone.0347776.s004.tiff]

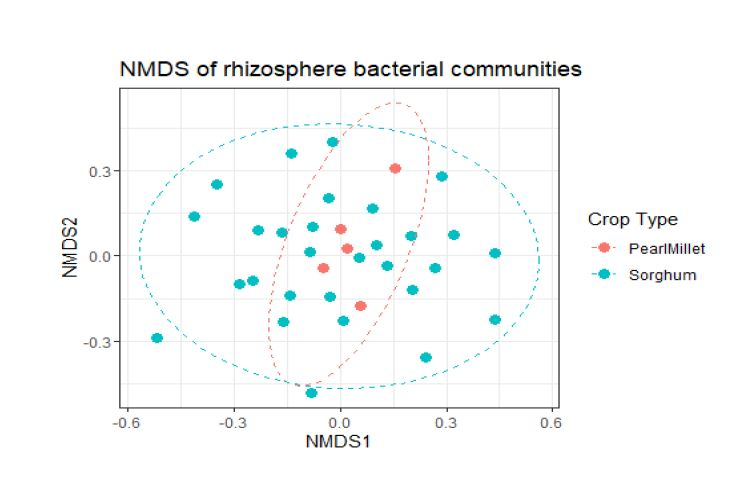

Supplement: S4 Fig — (TIFF) [file pone.0347776.s005.tiff]

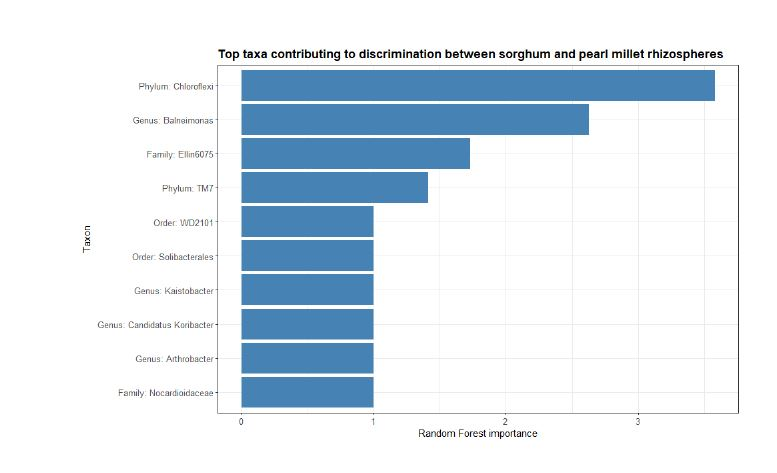

Supplement: S5 Fig — Importance values represent the mean decrease in classification accuracy when each taxon is removed from the model. (TIFF) [file pone.0347776.s006.tiff]
