# Supplementary material for: 16S rRNA-based metagenomics insights into the microbial diversity and functional attributes of soils from the rhizosphere of selected C4 crops of farms in Mpumalanga and Limpopo provinces, South Africa
Source: PLoS One. 2026 Jun 15;21(6):e0347776. doi: 10.1371/journal.pone.0347776 (PMC13268165; doi:10.1371/journal.pone.0347776)
Supplement: S1 Table — (DOCX) [file pone.0347776.s007.docx]

**S1 Table. Farm sites where soil samples were collected in the two provinces.**

| **Site** | **Province** | **GPS coordinates** | **Crop** | **Number of pooled samples** | **Number of representative samples** |
| --- | --- | --- | --- | --- | --- |
| Standerton farm 1 | Mpumalanga | 27^o^08'43.1''S2 9^o^27'21.4''E | Sorghum | 20 | 4 |
| Standerton farm 2 | Mpumalanga | 27^o^07'51.1''S 29^o^26'37.6''E | Sorghum | 10 | 2 |
| Standerton farm 3 | Mpumalanga | 27^o^08'03.9''S 29^o^27'28.2''E | Sorghum | 10 | 2 |
| Jane Furse, Maseleseleng farm 1 | Limpopo | 24^o^37'53.4''S 29^o^53'48.2''E | Sorghum | 5 | 1 |
| Jane Furse, Maseleseleng farm 2 | Limpopo | 24^o^38'00.7''S 29^o^53'45.1''E | Sorghum | 30 | 6 |
| Jane Furse, Maseleseleng farm 3 | Limpopo | 24^o^37'59.6''S 29^o^53'44.9''E | Sorghum | 25 | 5 |
| Jane Furse, Maseleseleng farm 4 | Limpopo | 24^o^37'49.1''S 29^o^54'25.6''E | Sorghum | 15 | 3 |
| Lebowakgomo farm 1 | Limpopo | 24^o^16'24.5''S 29^o^33'18.7''E | Sorghum | 5 | 1 |
| Lebowakgomo farm 2 | Limpopo | 24^o^16'21.0''S 29^o^33'09.0''E | Pearl millet | 10 | 2 |
| Lebowakgomo farm 3 | Limpopo | 24o16'23.5''S 29^o^33'08.3''E | Pearl millet | 10 | 2 |
| Lebowakgomo farm 4 | Limpopo | 24o16'29.9''S 29^o^33'15.2''E | Sorghum | 5 | 1 |
| Lebowakgomo farm 5 | Limpopo | 24^o^16'29.9''S 29^o^33'15.2''E | Sorghum | 5 | 1 |
| Lebowakgomo farm 6 | Limpopo | 24^o^16'41.8''S 29^o^33'15.1''E | Sorghum | 5 | 1 |
| Lebowakgomo farm 7 | Limpopo | 24^o^16'41.4''S 29^o^33'16.9''E | Sorghum | 5 | 1 |
| Lebowakgomo farm 8 | Limpopo | 24^o^16'40.1''S 29^o^33'25.1''E | Pearl millet | 5 | 1 |
| Lebowakgomo farm 9 | Limpopo | 24^o^16'56.1''S 29^o^33'21.9''E | Sorghum | 5 | 1 |
