# Supplementary material for: 16S rRNA-based metagenomics insights into the microbial diversity and functional attributes of soils from the rhizosphere of selected C4 crops of farms in Mpumalanga and Limpopo provinces, South Africa
Source: PLoS One. 2026 Jun 15;21(6):e0347776. doi: 10.1371/journal.pone.0347776 (PMC13268165; doi:10.1371/journal.pone.0347776)
Supplement: S2 Table — (DOCX) [file pone.0347776.s008.docx]

**S2 Table.  Physical and chemical properties of soil samples from Standerton farms**

| **Analyte** | **Method** | **Units** | **Stand-Sor1A** | **Stand-Sor1B** | **Stand-Sor1C** | **Stand-Sor1D** | **Stand-Sor2A** | **Stand-Sor2B** | **Stand-Mai3A** | **Stand-Mai3B** |
| --- | --- | --- | --- | --- | --- | --- | --- | --- | --- | --- |
| Al | Tritrible acidity |  | 0 | 0 | 0 | 0 | 0.01 | 0 | 0 | 0 |
| Cu | HCl extract | mg/kg | 1.39 | 1.21 | 1.02 | 0.896 | 1.16 | 1.3 | 1.22 | 1.57 |
| Fe | HCl extract | mg/kg | 20.3 | 17.5 | 18 | 15 | 22.6 | 28.3 | 35.9 | 40.5 |
| Mn | HCl extract | mg/kg | 52.1 | 53.8 | 58.2 | 66.2 | 73.2 | 57.7 | 58.8 | 70.3 |
| NO3--N | KCl extract | mg/kg | 54.7 | 66 | 63.3 | 49.5 | 79.1 | 58.2 | 44.9 | 48 |
| P (Bray No. 1 or 2) | Bray 1 | mg/kg | 46.24 | 37.02 | 81.8 | 62.24 | 72.63 | 66.88 | 70.74 | 52.71 |
| Zn | HCl extract | mg/kg | 7.71 | 6.75 | 11.8 | 10.2 | 9.91 | 10.4 | 9.71 | 9.83 |
| Ca | Amm. Acetate | mg/kg | 197 | 199 | 186 | 200 | 155 | 132 | 147 | 115 |
| Mg | Amm. Acetate | mg/kg | 45.8 | 46.8 | 39.4 | 42.5 | 26 | 18.6 | 11.1 | 13.8 |
| Na | Amm. Acetate | mg/kg | 2.1 | 1.78 | 1.15 | 1.26 | 1.15 | 1.1 | 0.71 | 0.64 |
| K | Amm. Acetate | mg/kg | 26.4 | 27.7 | 34.5 | 35.7 | 36.4 | 36.5 | 27.8 | 31.2 |
| CEC | Titaration | cmlo+/kg | 17.85 | 18.14 | 15.69 | 15.27 | 11.85 | 4.19 | 7.33 | 9.47 |
| pH | water | * | 6.4 | 5.97 | 6.01 | 5.34 | 5.68 | 6.01 | 5.44 | 5.98 |
| Clay | Hydrometer | % | 36 | 36 | 36 | 36 | 30 | 26 | 16 | 20 |
| Sand | Hydrometer | % | 38 | 44 | 46 | 42 | 52 | 56 | 72 | 68 |
| Silt | Hydrometer | % | 26 | 20 | 18 | 22 | 18 | 18 | 12 | 12 |
| Soil textural class |  |  | Sandy clay | Sandy clay | Sandy clay | Sandy clay | Sandy clay loam | Sandy clay loam | Sandy clay loam | Sandy clay loam |
