# Supplementary material for: 16S rRNA-based metagenomics insights into the microbial diversity and functional attributes of soils from the rhizosphere of selected C4 crops of farms in Mpumalanga and Limpopo provinces, South Africa
Source: PLoS One. 2026 Jun 15;21(6):e0347776. doi: 10.1371/journal.pone.0347776 (PMC13268165; doi:10.1371/journal.pone.0347776)
Supplement: S3 Table — (DOCX) [file pone.0347776.s009.docx]

**S3 Table. Physical and chemical properties of soil samples from Jane Furse farms**

| **Analyte** | **Method** | **Units** | **Mase-Sor1A** | **Mase-Sor2A** | **Mase-Sor2B** | **Mase-Sor2C** | **Mase-Sor2D** | **Mase-Sor2E** | **Mase-Sor2F** | **Mase-Sor3A** | **Mase-Sor3B** | **Mase-Sor3C** | **Mase-Sor3D** | **Mase-Sor3E** | **Math-Sor1A** | **Math-Sor1B** | **Math-Sor1C** |
| --- | --- | --- | --- | --- | --- | --- | --- | --- | --- | --- | --- | --- | --- | --- | --- | --- | --- |
| Al | Tritrible acidity |  | 0 | 0 | 0 | 0 | 0 | 0 | 0 | 0 | 0 | 0 | 0 | 0 | 0 | 0 | 0 |
| Cu | HCl extract | mg/kg | 0.167 | 1.17 | 1.41 | 0.918 | 1.26 | 0.859 | 1.09 | 0.641 | 1.1 | 1.05 | 1.01 | 0.915 | 0.644 | 0.494 | 0.661 |
| Fe | HCl extract | mg/kg | 0.875 | 13.5 | 13.7 | 12.9 | 20 | 22.1 | 19.9 | 23.9 | 13.2 | 15.9 | 18.1 | 16 | 22.2 | 23.8 | 30 |
| Mn | HCl extract | mg/kg | 25.5 | 37.8 | 44.7 | 29.2 | 37.4 | 26.3 | 35.2 | 27.4 | 34.9 | 34.9 | 36.4 | 25.1 | 41.3 | 26.7 | 27.5 |
| NO3--N | KCl extract | mg/kg | 3.33 | 1.46 | 18.31 | 10.93 | 8.85 | 8.45 | 8.85 | 7.47 | 12.81 | 13.39 | 12.82 | 9.7 | 6.18 | 7.36 | 4.63 |
| P (Bray No. 1 or 2) | Bray 1 | mg/kg | 12.87 | 4.23 | 6.86 | 10.23 | 15.9 | 11.83 | 6.85 | 36.87 | 5.37 | 6.59 | 23.8 | 14.23 | 25.19 | 46.56 | 34.79 |
| Zn | HCl extract | mg/kg | 1.37 | 1.56 | 1.84 | 1.29 | 1.45 | 1.12 | 1.64 | 1.71 | 1.12 | 1.18 | 1.88 | 1.19 | 2.92 | 3.57 | 2.59 |
| Ca | Amm. Acetate | mg/kg | 379 | 47.2 | 49.4 | 40.5 | 54.7 | 60.4 | 26.5 | 61 | 35.4 | 34.3 | 39.3 | 32.8 | 83.6 | 84.9 | 62 |
| Mg | Amm. Acetate | mg/kg | 20.1 | 10.2 | 11.6 | 9.2 | 12.3 | 12.2 | 6.66 | 12.8 | 8.13 | 8.24 | 8.78 | 7.35 | 9.93 | 11.9 | 10.3 |
| Na | Amm. Acetate | mg/kg | 1.02 | 0.93 | 0.8 | 0.62 | 0.82 | 0.57 | 0.62 | 0.71 | 0.58 | 0.55 | 0.6 | 0.6 | 0.61 | 0.51 | 0.59 |
| K | Amm. Acetate | mg/kg | 33 | 19.9 | 36.1 | 29.4 | 33.3 | 26.9 | 25.9 | 46.3 | 34.8 | 31.1 | 41.3 | 29.6 | 33.7 | 28.1 | 21.8 |
| CEC | Titaration | cmlo+/kg | 4.95 | 10.42 | 6.33 | 4.95 | 5.11 | 5.61 | 5.94 | 4.97 | 4.46 | 3.78 | 1.5 | 4.82 | 4.63 | 3.82 | 18.17 |
| pH | water | * | 6.54 | 5.86 | 5.7 | 5.48 | 6.08 | 6.11 | 5.98 | 5.47 | 6.17 | 5.36 | 5.67 | 5.91 | 5.82 | 6.04 | 6.11 |
| Clay | Hydrometer | % | 12 | 18 | 18 | 14 | 18 | 14 | 14 | 12 | 16 | 14 | 14 | 12 | 10 | 10 | 12 |
| Sand | Hydrometer | % | 82 | 78 | 74 | 80 | 76 | 80 | 84 | 86 | 78 | 82 | 82 | 86 | 88 | 86 | 86 |
| Silt | Hydrometer | % | 6 | 4 | 8 | 6 | 6 | 6 | 2 | 2 | 6 | 4 | 4 | 2 | 2 | 4 | 2 |
| Soil textural class |  |  | Sandy Loam | Sandy Loam | Sandy Loam | Sandy Loam | Sandy Loam | Sandy Loam | Sandy loam | Sandy Loam | Sandy Loam | Sandy Loam | Sandy Loam | Sandy Loam | Sandy Loam | Sandy Loam | Sandy Loam |
