# Supplementary material for: 16S rRNA-based metagenomics insights into the microbial diversity and functional attributes of soils from the rhizosphere of selected C4 crops of farms in Mpumalanga and Limpopo provinces, South Africa
Source: PLoS One. 2026 Jun 15;21(6):e0347776. doi: 10.1371/journal.pone.0347776 (PMC13268165; doi:10.1371/journal.pone.0347776)
Supplement: S4 Table — (DOCX) [file pone.0347776.s010.docx]

**S4 Table. Physical and chemical properties of soil samples from Lebowakgomo farms**

| **Analyte** | **Method** | **Units** | **Lebo-Mill2A** | **Lebo-Mill2B** | **Lebo-Mill3A** | **Lebo-Mill3B** | **Lebo-Mill8** | **Lebo-Sor1** | **Lebo-Sor4** | **Lebo-Sor5** | **Lebo-Sor6** | **Lebo-Sor7** | **Lebo-Sor9** |
| --- | --- | --- | --- | --- | --- | --- | --- | --- | --- | --- | --- | --- | --- |
| Al | Tritrible acidity |  | 0 | 0 | 0 | 0 | 0 | 0,01 | 0 | 0 | 0 | 0 | 0 |
| Cu | HCl extract | mg/kg | 1,41 | 2,03 | 2,68 | 3,39 | 2,38 | 0,687 | 2,56 | 2,11 | 2,06 | 3,19 | 2,51 |
| Fe | HCl extract | mg/kg | 3,14 | 4,39 | 4,88 | 6,31 | 6,57 | 0,577 | 5,4 | 8,37 | 7,53 | 9,01 | 13 |
| Mn | HCl extract | mg/kg | 57,8 | 46,5 | 50,4 | 70,1 | 55,1 | 41,6 | 63,3 | 52,9 | 65,3 | 42,4 | 30,1 |
| NO3--N | KCl extract | mg/kg | 31,8 | 16,13 | 8,25 | 18,58 | 16,91 | 12,8 | 23,3 | 20,48 | 16,67 | 14,88 | 9,49 |
| P (Bray No. 1 or 2) | Bray 1 | mg/kg | 2,8 | 1,63 | 1,87 | 4,9 | 2,57 | 1,98 | 1,26 | 3,82 | 4,34 | 1,66 | 1,16 |
| Zn | HCl extract | mg/kg | 0,687 | 0,71 | 0,59 | 0,866 | 0,834 | 0,41 | 0,65 | 0,573 | 0,585 | 0,561 | 0,526 |
| Ca | Amm. Acetate | mg/kg | 267 | 176 | 172 | 159 | 150 | 415 | 241 | 134 | 121 | 128 | 74 |
| Mg | Amm. Acetate | mg/kg | 56,5 | 56,4 | 68,7 | 55,1 | 92,3 | 79,1 | 77,5 | 39,7 | 35,2 | 55,9 | 33,7 |
| Na | Amm. Acetate | mg/kg | 1,27 | 0,92 | 1,63 | 1,07 | 1,96 | 3,35 | 1,52 | 1,09 | 0,88 | 1,56 | 1,33 |
| K | Amm. Acetate | mg/kg | 58,4 | 38,8 | 33 | 45,4 | 39,3 | 24,9 | 32,1 | 38,4 | 34 | 29,1 | 20,2 |
| CEC | Titaration | cmlo+/kg | 14,69 | 17,59 | 16,55 | 21,34 | 16,74 | 18,36 | 12,33 | 12,71 | 13,34 | 18,56 | 11,38 |
| pH | water | * | 5,34 | 5,47 | 6,05 | 5,97 | 6,18 | 5,97 | 6,31 | 5,67 | 5,83 | 6,42 | 5,99 |
| Clay | Hydrometer | % | 36 | 36 | 38 | 30 | 36 | 38 | 40 | 28 | 26 | 28 | 22 |
| Sand | Hydrometer | % | 52 | 50 | 48 | 58 | 50 | 46 | 44 | 62 | 64 | 60 | 68 |
| Silt | Hydrometer | % | 12 | 14 | 14 | 12 | 14 | 16 | 16 | 10 | 10 | 12 | 10 |
| Soil textural class |  |  | Sandy clay | Sandy clay | Sandy clay | Sandy clay loam | Sandy clay | Sandy clay | clay loam | Sandy clay loam | Sandy clay loam | Sandy clay loam | Sandy clay loam |
