# Supplementary material for: 16S rRNA-based metagenomics insights into the microbial diversity and functional attributes of soils from the rhizosphere of selected C4 crops of farms in Mpumalanga and Limpopo provinces, South Africa
Source: PLoS One. 2026 Jun 15;21(6):e0347776. doi: 10.1371/journal.pone.0347776 (PMC13268165; doi:10.1371/journal.pone.0347776)
Supplement: Attachment — Submitted filename: Response to Reviewers.docx [file pone.0347776.s012.docx]

**Response to Reviewers**

Thank you for giving us the opportunity to submit a revised draft of the manuscript for publication. We have incorporated the suggestions made by the reviewers. Those changes are highlighted within the manuscript. Below are corrections and additional information included in the manuscript.

**Reviewers' Comments to the Authors:**

**Reviewer #1**:

1. **The study uses 16S rRNA amplicon sequencing, not shotgun metagenomics. Please revise the title, abstract, and throughout the text to avoid misleading terminology. Suggested alternative: “Amplicon-based insights into microbial diversity…”.**

**Author's response**: Thank you for the suggestion. The title has now been revised to ‘16S rRNA-based metagenomics insights into the microbial diversity and functional attributes of soils from the rhizosphere of selected C4 crops in Mpumalanga and Limpopo farms, South Africa’. Metagenomics has been revised to 16S rRNA amplicon sequencing throughout the script.

2. **The title and abstract indicate that samples were collected from the rhizospheres of both sorghum (Sorghum bicolor L.) and pearl millet (Pennisetum glaucum L.). However, in the Materials and Methods section (L118–135) and throughout the Results, there is no clear breakdown of how many samples were collected from sorghum fields and how many from pearl millet fields. It also remains unclear whether these were mono-cropped fields or combined cropping systems.**

**Author's response:** Thank you for pointing this out. The fields were monocropped, not combined cropping systems.

**i) How many samples came from each crop**

**Author's response:** It is now clarified in text that three samples were collected from pearl millet farms, while thirteen samples were collected from sorghum farms, also indicated in table S1.

**ii) Whether sorghum and millet rhizospheres were analysed separately or pooled,**

**Author's respons**e: They were analysed separately per crop and per field. It is now clarified in text and shown in table S1.

**(iii) whether differences between the two crops were tested statistically**

**Author's response:** The aim of this study was to analyze how soil physicochemical properties influence sorghum and pearl millet rhizosphere microbiomes in different locations of Mpumalanga and Limpopo provinces. Hence, statistical analyses to compare the crops were not performed.

3. **In L136–145, the authors list soil parameters analyzed but do not describe the analytical procedures or provide references for the methodologies used (e.g., pH determination, available P, exchangeable cations, nitrate, CEC, texture analysis). Since soil physicochemical parameters strongly influence microbial diversity, the manuscript must specify the exact analytical methods and cite appropriate references (e.g., AOAC, USDA, or FAO soil analysis protocols). Without this information, it is difficult to assess the reliability and comparability of the soil data.**

**Author's response:** Thank you for the comment. We agree that detailing the analytical procedures is essential for clarity and reproducibility. In the revised manuscript, we have now included a description of all soil physicochemical analyses conducted as well as the citation (handbook of standard soil testing methods, 1990).

4. **In Table 1, the authors present carbon substrate utilization patterns (carbohydrates, carboxylic acids, amino acids, polymers, amines), as well as AWCD, Shannon index (H), Richness (S), and Evenness (E). However, the Methods do not describe how substrate groups were derived from the Biolog EcoPlate assays, nor what statistical analyses were used to compare them. Please provide details on how the 31 carbon substrates were categorized and analyzed. Furthermore, in the Discussion, only AWCD and richness are briefly mentioned, but there is no interpretation of the substrate-level patterns. For example, differences in carbohydrate vs. amino acid utilization across farms could be linked to soil nutrient availability or microbial functional guilds. The authors should expand the discussion to integrate these functional diversity results with soil chemistry and sequencing data.**

**Author's response**: Thank you for highlighting the critical points regarding the Biolog EcoPlate data presented in Table 1. We have revised the Methods section and described how the 31 individual carbon substrates from the Biolog EcoPlate were categorized into the five functional groups presented in Table 1 (Carbohydrates, Carboxylic Acids, Amino Acids, Polymers, Amines). Statistical analysis of the biolog data is provided under the subheading Statistical analysis on community level physiological profile data and substrate level patterns has been included in the discussion section.

5. **In the Results (L350 onwards), the manuscript presents microbial distribution across soil samples at the phylum level (Fig. 1A–B). However, in the Methods section (L205–225), there is no description of how the phylum-level taxonomic summaries were generated. While the authors mention using Phyloseq and Vegan for alpha/beta diversity analyses and Galaxy for genus-level heatmaps, the pipeline for phylum-level relative abundance plots is not specified. Please clarify whether these phylum-level distributions were obtained via Phyloseq, QIIME2 taxa barplots, or another tool, and provide sufficient detail so the analysis is reproducible.**

**Author's response:** Thank you for pointing this out. We agree that the Methods section did not explicitly describe how the phylum-level taxonomic summaries were generated. We have now clarified this section and added full details to ensure reproducibility.

**6. The alpha and beta diversity analyses suffer from methodological and interpretational issues. For alpha diversity, it is not clear whether rarefaction or normalization was applied before calculating richness/diversity indices, which is essential for comparability across samples. Moreover, although the Kruskal–Wallis tests for alpha diversity were not significant (p > 0.34), the authors nonetheless describe Standerton as having higher diversity, which is misleading. For beta diversity, the PERMANOVA results are statistically significant but explain only 3–7% of variance, indicating very weak explanatory power. The NMDS ordination also shows considerable overlap. Therefore, ecological claims about strong site-level differences should be toned down.**

**Author's response**: Thank you for the comment. We agree with the misleading sentence about Standerton as having higher diversity compared to the other two locations. It has been corrected accordingly.

**7. Most importantly, the manuscript does not link alpha or beta diversity patterns to measured soil physicochemical variables. The study would be greatly strengthened by including correlation analyses (Spearman tests between soil properties and alpha indices; db-RDA/CCA linking soil parameters to beta diversity). This would provide real evidence for soil–microbiome interactions, rather than descriptive patterns.**

**Author's response:** Thank you for the comment. Spearman tests between soil properties and alpha indices; db-RDA linking soil parameters to beta diversity has been added to the result and discussion section (Figure 6A and 6B).

**8. While the chemical and physical properties of soils from the 16 farms (34 pooled samples) are well described (Tables S2–S4), the manuscript does not sufficiently integrate these data with the microbiome results. For example, alpha diversity patterns (Standerton > Lebowakgomo > Jane Furse) are discussed only in terms of pH, but no statistical correlations (e.g., Spearman correlations, CCA/RDA, Mantel tests) are provided to link soil variables with microbial richness, abundance, or community structure. This is a major gap, since the stated objective was to understand how soil physicochemical properties influence rhizosphere microbiomes. I strongly recommend conducting correlation analyses between soil parameters and microbial diversity metrics, and presenting multivariate plots (CCA, db-RDA, or redundancy analysis) to clearly demonstrate soil–microbiome relationships.**

**Author's response:** Thank you. Advice acknowledged and incorporated in the manuscript.

9. **The manuscript attributes ecological functions to phyla (e.g., Planctomycetes in nutrient cycling, Actinobacteria in biofertilization), but these functions are not measured. Please reframe as “****potential functions based on known literature” unless functional prediction tools (PICRUSt2, FAPROTAX, Tax4Fun) are applied.**

**Author's response:** Thanks for the comment. We have thoroughly revised the manuscript, specifically in the Discussion sections, to reframe all statements regarding ecological roles. We have replaced assertive claims with conditional language to reflect that these are potential functions inferred from existing literature.

**10. While beneficial taxa are discussed, there is no mention of potential pathogenic groups in the rhizosphere (e.g., Ralstonia, Xanthomonas, Fusarium).**

**Author's response:** Thanks for the comment. The data presented in the current manuscript does not contain pathogenic bacteria as shown in Figures 1, 2 and 3. Our focus was on the most predominant phyla and genera in which no pathogen was detected.

**11. The manuscript refers to several supplementary items (Tables S1–S4, Figures S1–S3), but these were not included in the review package. Since these materials are critical for verifying sampling design, soil chemistry data, and diversity analyses, please ensure that all supplementary files are uploaded and available for reviewers and readers. Without them, it is not possible to confirm the validity of some of your results.**

**Author's response:** Thank you for bringing to our attention that the supplementary data ( Tables S1–S4, Figures S1–S3) were not included in the review package. We sincerely apologize for this critical oversight. We ensure that supplementary data is uploaded with the revised manuscript.

12. **Claims about “developing microbial bioinoculants” are speculative. Please tone down to “****providing baseline data that may inform future bioinoculant development.”**

**Author's response:** Thank you for the comment. We have toned down to ‘providing baseline data that may inform future bioinoculant development’.

**Reviewer #2:**

1. **Any justification for sampling soil at 20-30 cm soil depth for this microbial study while completely excluding the surface 0-15 cm depth, where there is an abundance of soil carbon?**

**Author's response:** Thank you for pointing this out. We acknowledge that the 20-30 cm depth was incorrectly stated in the manuscript. In practice, the 0-15 cm soil depth was not excluded, as soil samples were collected from 0-30 cm depth. The manuscript has been revised accordingly.
**2. Inconsistent numbering of tables in the text, which is further complicated by the unavailability of Tables S1 – S4. The same applies to the numbering of figures cited in the text (see: Fig.1 versus Fig. S1).**

**Author's response:** Thank you for pointing out the inconsistency. We have carefully revised the numbering of all tables and figures in both the main text and the supplementary material to ensure accuracy and consistency.

**3. Figure 1A in the main manuscript illustrates the relative abundance of the top 30 microbial phyla across the 34 soil samples. In contrast, Figure S1, which is included in the supplementary material, presents the dendrogram showing dissimilarities in carbon utilization patterns. These corrections have now been incorporated, and all in-text citations have been updated accordingly.**

**Author's response:** We have ensured that supplementary data is uploaded with the revised manuscript.

4. **Figure 4 on Alpha diversity measures by type is dull; please provide a brighter and clearer picture**

**Author's response:** Thank you for the comment. Figure 4 has been updated with a brighter and clearer image

5. **The units of measurement for the various C-sources (i.e., carbohydrates, carboxylic acids, amino acids, polymers, and amines) presented in Table 1 are missing!**

**Author's response:** Thank you for the comment. We have clarified in Table 1 keynotes that carbon-source utilization values are expressed as optical density readings at 590 nm (OD_590_).

6. **Over and above the indication of the percent composition of the primary mineral composition of the soils tested, the textural class for each soil must be provided for ease of communication and management implications.**

**Author's response:** Thank you for the comment. Soil textural class of each soil sample has been provided in the result section and in Table S2 – S4.

7. **Edit the entire manuscript to avoid repetition, e.g., average well-color development (AWCD)**

**Author's response:** Thank you for the comment. The manuscript has been revised to remove repetitions and improve clarity.
