# Supplementary material for: 16S rRNA-based metagenomics insights into the microbial diversity and functional attributes of soils from the rhizosphere of selected C4 crops of farms in Mpumalanga and Limpopo provinces, South Africa
Source: PLoS One. 2026 Jun 15;21(6):e0347776. doi: 10.1371/journal.pone.0347776 (PMC13268165; doi:10.1371/journal.pone.0347776)
Supplement: Attachment — Submitted filename: Response to Reviewers_R2.docx [file pone.0347776.s013.docx]

**Response to Reviewers**

Thank you for giving us the opportunity to submit a revised draft of the manuscript (R2) for consideration to be published on PLOS One. We have made every effort to incorporate the suggestions made by the reviewers, in which all the changes made are highlighted in the main manuscript. We have also submitted clear unmarked version of the edited manuscript.

1. Reviewer’s comment.

You have identified Mn, Fe, NO₃-N, and Ca as significant drivers of microbial community structure and functional diversity. Currently, these are treated as inherent properties of the location. The missing link are these minerals a result of soil parent material or human intervention. For example, high NO₃-N levels typically suggest synthetic nitrogen fertilization or manure application. High Ca levels are often a fingerprint of liming practices used to manage soil pH.
In the Discussion, please interpret these findings through an agronomic lens. Discuss whether these "key drivers" reflect the cultural practices (fertilization, liming, tillage) common to Mpumalanga and Limpopo. This will transform your results into actionable insights for soil health management.

**Author’s Response:** We thank the reviewer for raising this important point. Discussion has been added to interpret the findings through an agronomic lens.

2. Reviewer’s comment

The comparison between sorghum and pearl millet rhizospheres is currently inexplicit. As these are both C4 grasses, their rhizosphere signatures may be similar, but this must be addressed directly. It is unclear if "Crop Type" was a significant factor in your PERMANOVA. If there was no difference, you must discuss why (e.g., is the "Soil Effect" simply stronger than the "Host Effect"?).
Please explicitly state the statistical difference (or lack thereof) between the two crops. I suggest using a discriminant analysis (such as LEfSe or Random Forest) to identify specific "indicator taxa" that might be unique to each crop, even if the overall community structure looks similar.

**Author’s Response:** We thank the reviewer for raising this important point.

To determine whether host crop identity influenced rhizosphere bacterial communities, we performed PERMANOVA analysis based on Bray–Curtis dissimilarity. The analysis showed **that** crop type (sorghum vs pearl millet) did not significantly influence rhizosphere bacterial community composition (PERMANOVA, R² = 0.031, p = 0.286).

When site was included in the model, location had a significant effect on microbial community structure (PERMANOVA, R² = 0.074, p = 0.001), whereas crop type remained non-significant (R² = 0.032, p = 0.117). These results indicate that site-associated environmental variation exerted a stronger influence on rhizosphere bacterial community composition than host plant identity.

Consistent with these results, NMDS ordination based on Bray–Curtis dissimilarity showed substantial overlap between sorghum and pearl millet rhizosphere samples, suggesting limited separation of bacterial communities between the two crop types.

Random Forest analysis identified several taxa contributing to discrimination between sorghum and pearl millet rhizosphere samples, including members of Chloroflexi, Balneimonas, Ellin6075, TM7, and Solibacterales. However, the importance values were generally low, indicating limited discriminatory power between the two crop rhizospheres, which is consistent with the PERMANOVA results showing no significant crop-type effect on overall microbial community composition.

3. Reviewer’s comment

The manuscript frequently cites "geographic variation" between the two provinces, but the reader has no way to visualize or verify this. Include a map of the 16 sampling sites across Mpumalanga and Limpopo as a main figure. This helps clarify the spatial scale of your study.
Provide a summary meteorological data as table (or include in the site description) of the mean annual precipitation (MAP) and temperature for the sampling period. Soil microbes are highly sensitive to moisture and temperature; without this data, "geographic variation" remains an undefined variable.

**Author's response:** Thank you for pointing this out. A site description including sampling period, temperature and annual precipitation has been added under site and soil collection sub-heading, under method and material.

4. Reviewer’s comment

The Biolog results show variations in carbon source utilization (e.g., carbohydrates vs. carboxylic acids). Link these functional shifts back to the soil chemistry. For instance, does higher nitrate availability correlate with the utilization of specific amino acids? Linking the Chemical (Soil), Biological (16S), and Functional (Biolog) datasets more tightly will provide a more holistic view of the rhizosphere ecosystem.

**Author’s response**: Thank you for the comment: We have incorporated a correlation analysis into the study to support and tighten our discussion on rhizosphere ecosystem. This analysis is included in result and discussion section.

5. Reviewers comment

Please provide a brief description of the farming systems. Were these subsistence farms? Were they under conventional tillage or conservation agriculture? This context is vital for interpreting the "intensive farming" claims made in your introduction. Your models explain a specific percentage of the variance (e.g., 7.38%). Please discuss what might account for the "unexplained" variance (e.g., unmeasured factors like soil moisture at sampling or pesticide history).
The technical foundation of your revision is strong. By adding the geographic and agronomic context requested above, you will ensure that your research provides a meaningful contribution to our understanding of C4 crop-microbe interactions in South African smallholder systems. I look forward to seeing these improvements in the next version.

**Author’s response:** Thank you for the comment: We have added the geographic and agronomic context in the manuscript and the unexplained variance has been discussed.
