# Supplementary material for: 16S rRNA-based metagenomics insights into the microbial diversity and functional attributes of soils from the rhizosphere of selected C4 crops of farms in Mpumalanga and Limpopo provinces, South Africa
Source: PLoS One. 2026 Jun 15;21(6):e0347776. doi: 10.1371/journal.pone.0347776 (PMC13268165; doi:10.1371/journal.pone.0347776)
Supplement: Peer review history

# Peer review: Decision Letter 0

**DOI:** 10.1371/journal.pone.0347776.r001

**Authors:** Ying Ma

28 Oct 2025

PONE-D-25-48417

Metagenomic insights into the microbial and functional diversity of soils from the rhizosphere of sorghum (Sorghum bicolor L.) and pearl millet ( Pennisetum glaucum L.) on selected farms in South Africa

PLOS ONE

Dear Dr. Hassen,

Thank you for submitting your manuscript to PLOS ONE. After careful consideration, we feel that it has merit but does not fully meet PLOS ONE’s publication criteria as it currently stands. Therefore, we invite you to submit a revised version of the manuscript that addresses the points raised during the review process.

Please submit your revised manuscript by Dec 12 2025 11:59PM. If you will need more time than this to complete your revisions, please reply to this message or contact the journal office at plosone@plos.org. When you're ready to submit your revision, log on to https://www.editorialmanager.com/pone/ and select the 'Submissions Needing Revision' folder to locate your manuscript file.

Please include the following items when submitting your revised manuscript:

- A rebuttal letter that responds to each point raised by the academic editor and reviewer(s). You should upload this letter as a separate file labeled 'Response to Reviewers'.
- A marked-up copy of your manuscript that highlights changes made to the original version. You should upload this as a separate file labeled 'Revised Manuscript with Track Changes'.
- An unmarked version of your revised paper without tracked changes. You should upload this as a separate file labeled 'Manuscript'.

If you would like to make changes to your financial disclosure, please include your updated statement in your cover letter. Guidelines for resubmitting your figure files are available below the reviewer comments at the end of this letter.

If applicable, we recommend that you deposit your laboratory protocols in protocols.io to enhance the reproducibility of your results. Protocols.io assigns your protocol its own identifier (DOI) so that it can be cited independently in the future. For instructions see: https://journals.plos.org/plosone/s/submission-guidelines#loc-laboratory-protocols. Additionally, PLOS ONE offers an option for publishing peer-reviewed Lab Protocol articles, which describe protocols hosted on protocols.io. Read more information on sharing protocols at https://plos.org/protocols?utm_medium=editorial-email&utm_source=authorletters&utm_campaign=protocols.

We look forward to receiving your revised manuscript.

Kind regards,

Ying Ma, Ph.D.

Academic Editor

PLOS ONE

Journal Requirements:

When submitting your revision, we need you to address these additional requirements.

1. Please ensure that your manuscript meets PLOS ONE's style requirements, including those for file naming. The PLOS ONE style templates can be found at

https://journals.plos.org/plosone/s/file?id=wjVg/PLOSOne_formatting_sample_main_body.pdf and

https://journals.plos.org/plosone/s/file?id=ba62/PLOSOne_formatting_sample_title_authors_affiliations.pdf.

2. In your Methods section, please provide additional information regarding the permits you obtained for the work. Please ensure you have included the full name of the authority that approved the field site access and, if no permits were required, a brief statement explaining why.

3. Thank you for stating the following financial disclosure:

“The funding for this project was obtained from the Department of Agriculture (DoA) of South Africa under funding number A-134”

Please state what role the funders took in the study.  If the funders had no role, please state: "The funders had no role in study design, data collection and analysis, decision to publish, or preparation of the manuscript."

If this statement is not correct you must amend it as needed.

Please include this amended Role of Funder statement in your cover letter; we will change the online submission form on your behalf.

4. Thank you for stating the following in the Acknowledgments Section of your manuscript:

“We thank the Department of Agriculture (DoA) of South Africa for funding this project. MOD-M and TM express their gratitude to the National Research Foundation (NRF) of South Africa for Postdoctoral Fellowships under the ARC-PDP block grant for postgraduate programme (PDP/2023/04/03/02).”

We note that you have provided additional information within the Acknowledgements Section that is not currently declared in your Funding Statement. Please note that funding information should not appear in the Acknowledgments section or other areas of your manuscript. We will only publish funding information present in the Funding Statement section of the online submission form.

Please remove any funding-related text from the manuscript and let us know how you would like to update your Funding Statement. Currently, your Funding Statement reads as follows:

“The funding for this project was obtained from the Department of Agriculture (DoA) of South Africa under funding number A-134”

Please include your amended statements within your cover letter; we will change the online submission form on your behalf.

5. Please include a copy of Table 2 which you refer to in your text on page 6.

6. If the reviewer comments include a recommendation to cite specific previously published works, please review and evaluate these publications to determine whether they are relevant and should be cited. There is no requirement to cite these works unless the editor has indicated otherwise.

[Note: HTML markup is below. Please do not edit.]

Reviewers' comments:

Reviewer's Responses to Questions

**Comments to the Author**

1. Is the manuscript technically sound, and do the data support the conclusions?

The manuscript must describe a technically sound piece of scientific research with data that supports the conclusions. Experiments must have been conducted rigorously, with appropriate controls, replication, and sample sizes. The conclusions must be drawn appropriately based on the data presented.

Reviewer #1: Partly

Reviewer #2: Yes

**********

2. Has the statistical analysis been performed appropriately and rigorously?

Reviewer #1: No

Reviewer #2: Yes

**********

3. Have the authors made all data underlying the findings in their manuscript fully available?

The PLOS Data policy requires authors to make all data underlying the findings described in their manuscript fully available without restriction, with rare exception (please refer to the Data Availability Statement in the manuscript PDF file). The data should be provided as part of the manuscript or its supporting information, or deposited to a public repository. For example, in addition to summary statistics, the data points behind means, medians and variance measures should be available. If there are restrictions on publicly sharing data—e.g. participant privacy or use of data from a third party—those must be specified.

Reviewer #1: No

Reviewer #2: No

**********

4. Is the manuscript presented in an intelligible fashion and written in standard English?

PLOS ONE does not copyedit accepted manuscripts, so the language in submitted articles must be clear, correct, and unambiguous. Any typographical or grammatical errors should be corrected at revision, so please note any specific errors here.

Reviewer #1: Yes

Reviewer #2: Yes

**********

5. Review Comments to the Author

Please use the space provided to explain your answers to the questions above. You may also include additional comments for the author, including concerns about dual publication, research ethics, or publication ethics. (Please upload your review as an attachment if it exceeds 20,000 characters)

Reviewer #1: The manuscript “Metagenomic insights into the microbial and functional diversity of soils from the rhizosphere of sorghum and pearl millet on selected farms in South Africa” addresses an important and underexplored area: the microbial ecology of sorghum and pearl millet rhizospheres in South Africa. The combined use of soil physicochemical analyses, Biolog EcoPlate (CLPP), and 16S rRNA sequencing provides a valuable dataset. The work has potential significance for sustainable agriculture and bioinoculant development.

However, the manuscript requires substantial revision before it can be considered for publication. There are issues with methodology clarity, data integration, interpretation, and missing supplementary information. Below, I provide specific comments.

• The study uses 16S rRNA amplicon sequencing, not shotgun metagenomics. Please revise the title, abstract, and throughout the text to avoid misleading terminology. Suggested alternative: “Amplicon-based insights into microbial diversity…”.

• The title and abstract indicate that samples were collected from the rhizospheres of both sorghum (Sorghum bicolor L.) and pearl millet (Pennisetum glaucum L.). However, in the Materials and Methods section (L118–135) and throughout the Results, there is no clear breakdown of how many samples were collected from sorghum fields and how many from pearl millet fields. It also remains unclear whether these were mono-cropped fields or combined cropping systems. Furthermore, the Results and Discussion do not present any direct comparison between the rhizosphere microbiomes of sorghum vs. pearl millet. The authors must clarify: (i) how many samples came from each crop, (ii) whether sorghum and millet rhizospheres were analyzed separately or pooled, and (iii) whether differences between the two crops were tested statistically. Otherwise, the current title is misleading.

• In L136–145, the authors list soil parameters analyzed but do not describe the analytical procedures or provide references for the methodologies used (e.g., pH determination, available P, exchangeable cations, nitrate, CEC, texture analysis). Since soil physicochemical parameters strongly influence microbial diversity, the manuscript must specify the exact analytical methods and cite appropriate references (e.g., AOAC, USDA, or FAO soil analysis protocols). Without this information, it is difficult to assess the reliability and comparability of the soil data.

• While the chemical and physical properties of soils from the 16 farms (34 pooled samples) are well described (Tables S2–S4), the manuscript does not sufficiently integrate these data with the microbiome results. For example, alpha diversity patterns (Standerton > Lebowakgomo > Jane Furse) are discussed only in terms of pH, but no statistical correlations (e.g., Spearman correlations, CCA/RDA, Mantel tests) are provided to link soil variables with microbial richness, abundance, or community structure. This is a major gap, since the stated objective was to understand how soil physicochemical properties influence rhizosphere microbiomes. I strongly recommend conducting correlation analyses between soil parameters and microbial diversity metrics, and presenting multivariate plots (CCA, db-RDA, or redundancy analysis) to clearly demonstrate soil–microbiome relationships.

• In Table 1, the authors present carbon substrate utilization patterns (carbohydrates, carboxylic acids, amino acids, polymers, amines), as well as AWCD, Shannon index (H), Richness (S), and Evenness (E). However, the Methods do not describe how substrate groups were derived from the Biolog EcoPlate assays, nor what statistical analyses were used to compare them. Please provide details on how the 31 carbon substrates were categorized and analyzed. Furthermore, in the Discussion, only AWCD and richness are briefly mentioned, but there is no interpretation of the substrate-level patterns. For example, differences in carbohydrate vs. amino acid utilization across farms could be linked to soil nutrient availability or microbial functional guilds. The authors should expand the discussion to integrate these functional diversity results with soil chemistry and sequencing data.

• In the Results (L350 onwards), the manuscript presents microbial distribution across soil samples at the phylum level (Fig. 1A–B). However, in the Methods section (L205–225), there is no description of how the phylum-level taxonomic summaries were generated. While the authors mention using Phyloseq and Vegan for alpha/beta diversity analyses and Galaxy for genus-level heatmaps, the pipeline for phylum-level relative abundance plots is not specified. Please clarify whether these phylum-level distributions were obtained via Phyloseq, QIIME2 taxa barplots, or another tool, and provide sufficient detail so the analysis is reproducible.

• The alpha and beta diversity analyses suffer from methodological and interpretational issues. For alpha diversity, it is not clear whether rarefaction or normalization was applied before calculating richness/diversity indices, which is essential for comparability across samples. Moreover, although the Kruskal–Wallis tests for alpha diversity were not significant (p > 0.34), the authors nonetheless describe Standerton as having higher diversity, which is misleading. For beta diversity, the PERMANOVA results are statistically significant but explain only 3–7% of variance, indicating very weak explanatory power. The NMDS ordination also shows considerable overlap. Therefore, ecological claims about strong site-level differences should be toned down. Most importantly, the manuscript does not link alpha or beta diversity patterns to measured soil physicochemical variables. The study would be greatly strengthened by including correlation analyses (Spearman tests between soil properties and alpha indices; db-RDA/CCA linking soil parameters to beta diversity). This would provide real evidence for soil–microbiome interactions, rather than descriptive patterns.

• The manuscript attributes ecological functions to phyla (e.g., Planctomycetes in nutrient cycling, Actinobacteria in biofertilization), but these functions are not measured. Please reframe as “potential functions based on known literature” unless functional prediction tools (PICRUSt2, FAPROTAX, Tax4Fun) are applied.

• While beneficial taxa are discussed, there is no mention of potential pathogenic groups in the rhizosphere (e.g., Ralstonia, Xanthomonas, Fusarium). Screening for known plant pathogens would increase the applied relevance of the study.

• The manuscript refers to several supplementary items (Tables S1–S4, Figures S1–S3), but these were not included in the review package. Since these materials are critical for verifying sampling design, soil chemistry data, and diversity analyses, please ensure that all supplementary files are uploaded and available for reviewers and readers. Without them, it is not possible to confirm the validity of some of your results.

• Claims about “developing microbial bioinoculants” are speculative. Please tone down to “providing baseline data that may inform future bioinoculant development.”

Reviewer #2: The study investigated the bacterial microbiome diversity and abundance in 34 rhizosphere soil samples pooled from sixteen sorghum (Sorghum bicolor L.) and pearl millet (Pennisetum glaucum L.) farms in Limpopo and Mpumalanga. Below are gaps identified in the manuscript:

• Any justification for sampling soil at 20-30 cm soil depth for this microbial study while completely excluding the surface 0-15 cm depth, where there is an abundance of soil carbon?

• Inconsistent numbering of tables in the text, which is further complicated by the unavailability of Tables S1 – S4. The same applies to the numbering of figures cited in the text (see: Fig.1 versus Fig. S1).

• Figure 4 on Alpha diversity measures by type is dull; please provide a brighter and clearer picture

• The units of measurement for the various C-sources (i.e., carbohydrates, carboxylic acids, amino acids, polymers, and amines) presented in Table 1 are missing!

• Over and above the indication of the percent composition of the primary mineral composition of the soils tested, the textural class for each soil must be provided for ease of communication and management implications.

• Edit the entire manuscript to avoid repetition, e.g., average well-color development (AWCD)

**********

6. PLOS authors have the option to publish the peer review history of their article (what does this mean?). If published, this will include your full peer review and any attached files.

If you choose “no”, your identity will remain anonymous but your review may still be made public.

**Do you want your identity to be public for this peer review?**   For information about this choice, including consent withdrawal, please see our Privacy Policy.

Reviewer #1: **Yes:**  Dr Edwinraj Esack

Reviewer #2: No

**********

[NOTE: If reviewer comments were submitted as an attachment file, they will be attached to this email and accessible via the submission site. Please log into your account, locate the manuscript record, and check for the action link "View Attachments". If this link does not appear, there are no attachment files.]

To ensure your figures meet our technical requirements, please review our figure guidelines: https://journals.plos.org/plosone/s/figures

You may also use PLOS’s free figure tool, NAAS, to help you prepare publication quality figures: https://journals.plos.org/plosone/s/figures#loc-tools-for-figure-preparation.

NAAS will assess whether your figures meet our technical requirements by comparing each figure against our figure specifications.

# Author comment: Author response to Decision Letter 1

**DOI:** 10.1371/journal.pone.0347776.r002

23 Jan 2026

Response to Reviewers

Thank you for giving us the opportunity to submit a revised draft of the manuscript for publication. We have incorporated the suggestions made by the reviewers. Those changes are highlighted within the manuscript. Below are corrections and additional information included in the manuscript.

Reviewers' Comments to the Authors:

Reviewer #1:

1. The study uses 16S rRNA amplicon sequencing, not shotgun metagenomics. Please revise the title, abstract, and throughout the text to avoid misleading terminology. Suggested alternative: “Amplicon-based insights into microbial diversity…”.

Author's response: Thank you for the suggestion. The title has now been revised to ‘16S rRNA-based metagenomics insights into the microbial diversity and functional attributes of soils from the rhizosphere of selected C4 crops in Mpumalanga and Limpopo farms, South Africa’. Metagenomics has been revised to 16S rRNA amplicon sequencing throughout the script.

2. The title and abstract indicate that samples were collected from the rhizospheres of both sorghum (Sorghum bicolor L.) and pearl millet (Pennisetum glaucum L.). However, in the Materials and Methods section (L118–135) and throughout the Results, there is no clear breakdown of how many samples were collected from sorghum fields and how many from pearl millet fields. It also remains unclear whether these were mono-cropped fields or combined cropping systems.

Author's response: Thank you for pointing this out. The fields were monocropped, not combined cropping systems.

i) How many samples came from each crop?

Author's response: It is now clarified in text that three samples were collected from pearl millet farms, while thirteen samples were collected from sorghum farms, also indicated in table S1.

ii) Whether sorghum and millet rhizospheres were analysed separately or pooled,

Author's response: They were analysed separately per crop and per field. It is now clarified in text and shown in table S1.

(iii) whether differences between the two crops were tested statistically

Author's response: The aim of this study was to analyze how soil physicochemical properties influence sorghum and pearl millet rhizosphere microbiomes in different locations of Mpumalanga and Limpopo provinces. Hence, statistical analyses to compare the crops were not performed.

3. In L136–145, the authors list soil parameters analyzed but do not describe the analytical procedures or provide references for the methodologies used (e.g., pH determination, available P, exchangeable cations, nitrate, CEC, texture analysis). Since soil physicochemical parameters strongly influence microbial diversity, the manuscript must specify the exact analytical methods and cite appropriate references (e.g., AOAC, USDA, or FAO soil analysis protocols). Without this information, it is difficult to assess the reliability and comparability of the soil data.

Author's response: Thank you for the comment. We agree that detailing the analytical procedures is essential for clarity and reproducibility. In the revised manuscript, we have now included a description of all soil physicochemical analyses conducted as well as the citation (handbook of standard soil testing methods, 1990).

4. In Table 1, the authors present carbon substrate utilization patterns (carbohydrates, carboxylic acids, amino acids, polymers, amines), as well as AWCD, Shannon index (H), Richness (S), and Evenness (E). However, the Methods do not describe how substrate groups were derived from the Biolog EcoPlate assays, nor what statistical analyses were used to compare them. Please provide details on how the 31 carbon substrates were categorized and analyzed. Furthermore, in the Discussion, only AWCD and richness are briefly mentioned, but there is no interpretation of the substrate-level patterns. For example, differences in carbohydrate vs. amino acid utilization across farms could be linked to soil nutrient availability or microbial functional guilds. The authors should expand the discussion to integrate these functional diversity results with soil chemistry and sequencing data.

Author's response: Thank you for highlighting the critical points regarding the Biolog EcoPlate data presented in Table 1. We have revised the Methods section and described how the 31 individual carbon substrates from the Biolog EcoPlate were categorized into the five functional groups presented in Table 1 (Carbohydrates, Carboxylic Acids, Amino Acids, Polymers, Amines). Statistical analysis of the biolog data is provided under the subheading Statistical analysis on community level physiological profile data and substrate level patterns has been included in the discussion section.

5. In the Results (L350 onwards), the manuscript presents microbial distribution across soil samples at the phylum level (Fig. 1A–B). However, in the Methods section (L205–225), there is no description of how the phylum-level taxonomic summaries were generated. While the authors mention using Phyloseq and Vegan for alpha/beta diversity analyses and Galaxy for genus-level heatmaps, the pipeline for phylum-level relative abundance plots is not specified. Please clarify whether these phylum-level distributions were obtained via Phyloseq, QIIME2 taxa barplots, or another tool, and provide sufficient detail so the analysis is reproducible.

Author's response: Thank you for pointing this out. We agree that the Methods section did not explicitly describe how the phylum-level taxonomic summaries were generated. We have now clarified this section and added full details to ensure reproducibility.

6. The alpha and beta diversity analyses suffer from methodological and interpretational issues. For alpha diversity, it is not clear whether rarefaction or normalization was applied before calculating richness/diversity indices, which is essential for comparability across samples. Moreover, although the Kruskal–Wallis tests for alpha diversity were not significant (p > 0.34), the authors nonetheless describe Standerton as having higher diversity, which is misleading. For beta diversity, the PERMANOVA results are statistically significant but explain only 3–7% of variance, indicating very weak explanatory power. The NMDS ordination also shows considerable overlap. Therefore, ecological claims about strong site-level differences should be toned down.

Author's response: Thank you for the comment. We agree with the misleading sentence about Standerton as having higher diversity compared to the other two locations. It has been corrected accordingly.

7. Most importantly, the manuscript does not link alpha or beta diversity patterns to measured soil physicochemical variables. The study would be greatly strengthened by including correlation analyses (Spearman tests between soil properties and alpha indices; db-RDA/CCA linking soil parameters to beta diversity). This would provide real evidence for soil–microbiome interactions, rather than descriptive patterns.

Author's response: Thank you for the comment. Spearman tests between soil properties and alpha indices; db-RDA linking soil parameters to beta diversity has been added to the result and discussion section (Figure 6A and 6B).

8. While the chemical and physical properties of soils from the 16 farms (34 pooled samples) are well described (Tables S2–S4), the manuscript does not sufficiently integrate these data with the microbiome results. For example, alpha diversity patterns (Standerton > Lebowakgomo > Jane Furse) are discussed only in terms of pH, but no statistical correlations (e.g., Spearman correlations, CCA/RDA, Mantel tests) are provided to link soil variables with microbial richness, abundance, or community structure. This is a major gap, since the stated objective was to understand how soil physicochemical properties influence rhizosphere microbiomes. I strongly recommend conducting correlation analyses between soil parameters and microbial diversity metrics, and presenting multivariate plots (CCA, db-RDA, or redundancy analysis) to clearly demonstrate soil–microbiome relationships.

Author's response: Thank you. Advice acknowledged and incorporated in the manuscript.

9. The manuscript attributes ecological functions to phyla (e.g., Planctomycetes in nutrient cycling, Actinobacteria in biofertilization), but these functions are not measured. Please reframe as “potential functions based on known literature” unless functional prediction tools (PICRUSt2, FAPROTAX, Tax4Fun) are applied.

Author's response: Thanks for the comment. We have thoroughly revised the manuscript, specifically in the Discussion sections, to reframe all statements regarding ecological roles. We have replaced assertive claims with conditional language to reflect that these are potential functions inferred from existing literature.

10. While beneficial taxa are discussed, there is no mention of potential pathogenic groups in the rhizosphere (e.g., Ralstonia, Xanthomonas, Fusarium).

Author's response: Thanks for the comment. The data presented in the current manuscript does not contain pathogenic bacteria as shown in Figures 1, 2 and 3. Our focus was on the most predominant phyla and genera in which no pathogen was detected.

11. The manuscript refers to several supplementary items (Tables S1–S4, Figures S1–S3), but these were not included in the review package. Since these materials are critical for verifying sampling design, soil chemistry data, and diversity analyses, please ensure that all supplementary files are uploaded and available for reviewers and readers. Without them, it is not possible to confirm the validity of some of your results.

Author's response: Thank you for bringing to our attention that the supplementary data ( Tables S1–S4, Figures S1–S3) were not included in the review package. We sincerely apologize for this critical oversight. We ensure that supplementary data is uploaded with the revised manuscript.

12. Claims about “developing microbial bioinoculants” are speculative. Please tone down to “providing baseline data that may inform future bioinoculant development.”

Author's response: Thank you for the comment. We have toned down to ‘providing baseline data that may inform future bioinoculant development’.

Reviewer #2:

1. Any justification for sampling soil at 20-30 cm soil depth for this microbial study while completely excluding the surface 0-15 cm depth, where there is an abundance of soil carbon?

Author's response: Thank you for pointing this out. We acknowledge that the 20-30 cm depth was incorrectly stated in the manuscript. In practice, the 0-15 cm soil depth was not excluded, as soil samples were collected from 0-30 cm depth. The manuscript has been revised accordingly.

2. Inconsistent numbering of tables in the text, which is further complicated by the unavailability of Tables S1 – S4. The same applies to the numbering of figures cited in the text (see: Fig.1 versus Fig. S1).

Author's response: Thank you for pointing out the inconsistency. We have carefully revised the numbering of all tables and figures in both the main text and the supplementary material to ensure accuracy and consistency.

3. Figure 1A in the main manuscript illustrates the relative abundance of the top 30 microbial phyla across the 34 soil samples. In contrast, Figure S1, which is included in the supplementary material, presents the dendrogram showing dissimilarities in carbon utilization patterns. These corrections have now been incorporated, and all in-text citations have been updated accordingly.

Author's response: We have ensured that supplementary data is uploaded with the revised manuscript.

4. Figure 4 on Alpha diversity measures by type is dull; please provide a brighter and clearer picture.

Author's response: Thank you for the comment. Figure 4 has been updated with a brighter and clearer image

5. The units of measurement for the various C-sources (i.e., carbohydrates, carboxylic acids, amino acids, polymers, and amines) presented in Table 1 are missing!

Author's response: Thank you for the comment. We have clarified in Table 1 keynotes that carbon-source utilization values are expressed as optical density readings at 590 nm (OD590).

6. Over and above the indication of the percent composition of the primary mineral composition of the soils tested, the textural class for each soil must be provided for ease of communication and management implications.

Author's response: Thank you for the comment. Soil textural class of each soil sample has been provided in the result section and in Table S2 – S4.

7. Edit the entire manuscript to avoid repetition, e.g., average well-color development (AWCD)

Author's response: Thank you for the comment. The manuscript has been revised to remove repetitions and improve clarity.

Attachment Submitted filename: Response to Reviewers.docx

# Peer review: Decision Letter 1

**DOI:** 10.1371/journal.pone.0347776.r003

**Authors:** Ying Ma

25 Feb 2026

PONE-D-25-48417R1

16S rRNA based metagenomics insight into the microbial diversity and functional attributes of soils from the rhizosphere of selected C4 crops of farms in Mpumalanga and Limpopo provinces, South Africa

PLOS One

Dear Dr. Hassen,

Thank you for submitting your manuscript to PLOS ONE. After careful consideration, we feel that it has merit but does not fully meet PLOS ONE’s publication criteria as it currently stands. Therefore, we invite you to submit a revised version of the manuscript that addresses the points raised during the review process.

Please submit your revised manuscript by Apr 11 2026 11:59PM. If you will need more time than this to complete your revisions, please reply to this message or contact the journal office at plosone@plos.org. When you're ready to submit your revision, log on to https://www.editorialmanager.com/pone/ and select the 'Submissions Needing Revision' folder to locate your manuscript file.

Please include the following items when submitting your revised manuscript:

- A letter that responds to each point raised by the academic editor and reviewer(s). You should upload this letter as a separate file labeled 'Response to Reviewers'.
- A marked-up copy of your manuscript that highlights changes made to the original version. You should upload this as a separate file labeled 'Revised Manuscript with Track Changes'.
- An unmarked version of your revised paper without tracked changes. You should upload this as a separate file labeled 'Manuscript'.

-->If you would like to make changes to your financial disclosure, please include your updated statement in your cover letter. Guidelines for resubmitting your figure files are available below the reviewer comments at the end of this letter.

If applicable, we recommend that you deposit your laboratory protocols in protocols.io to enhance the reproducibility of your results. Protocols.io assigns your protocol its own identifier (DOI) so that it can be cited independently in the future. For instructions see: https://journals.plos.org/plosone/s/submission-guidelines#loc-laboratory-protocols. Additionally, PLOS ONE offers an option for publishing peer-reviewed Lab Protocol articles, which describe protocols hosted on protocols.io. Read more information on sharing protocols at https://plos.org/protocols?utm_medium=editorial-email&utm_source=authorletters&utm_campaign=protocols.

We look forward to receiving your revised manuscript.

Kind regards,

Ying Ma, Ph.D.

Academic Editor

PLOS One

Journal Requirements:

1. If the reviewer comments include a recommendation to cite specific previously published works, please review and evaluate these publications to determine whether they are relevant and should be cited. There is no requirement to cite these works unless the editor has indicated otherwise.

2. Please review your reference list to ensure that it is complete and correct. If you have cited papers that have been retracted, please include the rationale for doing so in the manuscript text, or remove these references and replace them with relevant current references. Any changes to the reference list should be mentioned in the rebuttal letter that accompanies your revised manuscript. If you need to cite a retracted article, indicate the article’s retracted status in the References list and also include a citation and full reference for the retraction notice.

[Note: HTML markup is below. Please do not edit.]

Reviewers' comments:

Reviewer's Responses to Questions

**Comments to the Author**

1. If the authors have adequately addressed your comments raised in a previous round of review and you feel that this manuscript is now acceptable for publication, you may indicate that here to bypass the “Comments to the Author” section, enter your conflict of interest statement in the “Confidential to Editor” section, and submit your "Accept" recommendation.

Reviewer #1: All comments have been addressed

**********

2. Is the manuscript technically sound, and do the data support the conclusions?

The manuscript must describe a technically sound piece of scientific research with data that supports the conclusions. Experiments must have been conducted rigorously, with appropriate controls, replication, and sample sizes. The conclusions must be drawn appropriately based on the data presented.

Reviewer #1: Yes

**********

3. Has the statistical analysis been performed appropriately and rigorously?

Reviewer #1: Yes

**********

4. Have the authors made all data underlying the findings in their manuscript fully available?

The PLOS Data policy requires authors to make all data underlying the findings described in their manuscript fully available without restriction, with rare exception (please refer to the Data Availability Statement in the manuscript PDF file). The data should be provided as part of the manuscript or its supporting information, or deposited to a public repository. For example, in addition to summary statistics, the data points behind means, medians and variance measures should be available. If there are restrictions on publicly sharing data—e.g. participant privacy or use of data from a third party—those must be specified.

Reviewer #1: Yes

**********

5. Is the manuscript presented in an intelligible fashion and written in standard English?

PLOS ONE does not copyedit accepted manuscripts, so the language in submitted articles must be clear, correct, and unambiguous. Any typographical or grammatical errors should be corrected at revision, so please note any specific errors here.

Reviewer #1: Yes

**********

6. Review Comments to the Author

Please use the space provided to explain your answers to the questions above. You may also include additional comments for the author, including concerns about dual publication, research ethics, or publication ethics. (Please upload your review as an attachment if it exceeds 20,000 characters)

Reviewer #1: Dear Authors,

Thank you for the detailed response to the previous round of reviews. The manuscript has improved significantly, particularly regarding the clarity of the metagenomic methodology and the integration of multivariate statistical tools (dbRDA and Spearman correlations).

However, to elevate this work from a descriptive survey to a robust scientific study with clear agricultural applications, several critical gaps must be addressed. Most notably, the "geographic variation" identified in the results needs to be substantiated with meteorological and spatial data, and the influence of soil minerals must be explicitly linked to agronomic practices.

Below are my comments intended to help you finalize this manuscript.

1. You have identified Mn, Fe, NO₃-N, and Ca as significant drivers of microbial community structure and functional diversity. Currently, these are treated as inherent properties of the location. The missing link are these minerals a result of soil parent material or human intervention? For example, high NO₃-N levels typically suggest synthetic nitrogen fertilization or manure application. High Ca levels are often a fingerprint of liming practices used to manage soil pH.

In the Discussion, please interpret these findings through an agronomic lens. Discuss whether these "key drivers" reflect the cultural practices (fertilization, liming, tillage) common to Mpumalanga and Limpopo. This will transform your results into actionable insights for soil health management.

2. The comparison between sorghum and pearl millet rhizospheres is currently inexplicit. As these are both C4 grasses, their rhizosphere signatures may be similar, but this must be addressed directly. It is unclear if "Crop Type" was a significant factor in your PERMANOVA. If there was no difference, you must discuss why (e.g., is the "Soil Effect" simply stronger than the "Host Effect"?).

Please explicitly state the statistical difference (or lack thereof) between the two crops. I suggest using a discriminant analysis (such as LEfSe or Random Forest) to identify specific "indicator taxa" that might be unique to each crop, even if the overall community structure looks similar.

3. The manuscript frequently cites "geographic variation" between the two provinces, but the reader has no way to visualize or verify this. Include a map of the 16 sampling sites across Mpumalanga and Limpopo as a main figure. This helps clarify the spatial scale of your study.

Provide a summary meteorological data as table (or include in the site description) of the mean annual precipitation (MAP) and temperature for the sampling period. Soil microbes are highly sensitive to moisture and temperature; without this data, "geographic variation" remains an undefined variable.

4. The Biolog results show variations in carbon source utilization (e.g., carbohydrates vs. carboxylic acids).

Link these functional shifts back to the soil chemistry. For instance, does higher nitrate availability correlate with the utilization of specific amino acids? Linking the Chemical (Soil), Biological (16S), and Functional (Biolog) datasets more tightly will provide a more holistic view of the rhizosphere ecosystem.

5. Please provide a brief description of the farming systems. Were these subsistence farms? Were they under conventional tillage or conservation agriculture? This context is vital for interpreting the "intensive farming" claims made in your introduction. Your models explain a specific percentage of the variance (e.g., 7.38%). Please discuss what might account for the "unexplained" variance (e.g., unmeasured factors like soil moisture at sampling or pesticide history).

The technical foundation of your revision is strong. By adding the geographic and agronomic context requested above, you will ensure that your research provides a meaningful contribution to our understanding of C4 crop-microbe interactions in South African smallholder systems. I look forward to seeing these improvements in the next version.

**********

7. PLOS authors have the option to publish the peer review history of their article (what does this mean?). If published, this will include your full peer review and any attached files.

If you choose “no”, your identity will remain anonymous but your review may still be made public.

**Do you want your identity to be public for this peer review?**   For information about this choice, including consent withdrawal, please see our Privacy Policy.

Reviewer #1: **Yes:**  Edwinraj Esack

**********

[NOTE: If reviewer comments were submitted as an attachment file, they will be attached to this email and accessible via the submission site. Please log into your account, locate the manuscript record, and check for the action link "View Attachments". If this link does not appear, there are no attachment files.]

To ensure your figures meet our technical requirements, please review our figure guidelines: https://journals.plos.org/plosone/s/figures

You may also use PLOS’s free figure tool, NAAS, to help you prepare publication quality figures: https://journals.plos.org/plosone/s/figures#loc-tools-for-figure-preparation.

NAAS will assess whether your figures meet our technical requirements by comparing each figure against our figure specifications.

# Author comment: Author response to Decision Letter 2

**DOI:** 10.1371/journal.pone.0347776.r004

5 Apr 2026

Response to Reviewers

Thank you for giving us the opportunity to submit a revised draft of the manuscript (R2) for consideration to be published on PLOS One. We have made every effort to incorporate the suggestions made by the reviewers, in which all the changes made are highlighted in the main manuscript. We have also submitted clear unmarked version of the edited manuscript.

1. Reviewer’s comment.

You have identified Mn, Fe, NO₃-N, and Ca as significant drivers of microbial community structure and functional diversity. Currently, these are treated as inherent properties of the location. The missing link are these minerals a result of soil parent material or human intervention. For example, high NO₃-N levels typically suggest synthetic nitrogen fertilization or manure application. High Ca levels are often a fingerprint of liming practices used to manage soil pH.

In the Discussion, please interpret these findings through an agronomic lens. Discuss whether these "key drivers" reflect the cultural practices (fertilization, liming, tillage) common to Mpumalanga and Limpopo. This will transform your results into actionable insights for soil health management.

Author’s Response: We thank the reviewer for raising this important point. Discussion has been added to interpret the findings through an agronomic lens.

2. Reviewer’s comment

The comparison between sorghum and pearl millet rhizospheres is currently inexplicit. As these are both C4 grasses, their rhizosphere signatures may be similar, but this must be addressed directly. It is unclear if "Crop Type" was a significant factor in your PERMANOVA. If there was no difference, you must discuss why (e.g., is the "Soil Effect" simply stronger than the "Host Effect"?). Please explicitly state the statistical difference (or lack thereof) between the two crops. I suggest using a discriminant analysis (such as LEfSe or Random Forest) to identify specific "indicator taxa" that might be unique to each crop, even if the overall community structure looks similar.

Author’s Response:

We thank the reviewer for raising this important point. To determine whether host crop identity influenced rhizosphere bacterial communities, we performed PERMANOVA analysis based on Bray–Curtis dissimilarity. The analysis showed that crop type (sorghum vs pearl millet) did not significantly influence rhizosphere bacterial community composition (PERMANOVA, R² = 0.031, p = 0.286). When site was included in the model, location had a significant effect on microbial community structure (PERMANOVA, R² = 0.074, p = 0.001), whereas crop type remained non-significant (R² = 0.032, p = 0.117). These results indicate that site-associated environmental variation exerted a stronger influence on rhizosphere bacterial community composition than host plant identity. Consistent with these results, NMDS ordination based on Bray–Curtis dissimilarity showed substantial overlap between sorghum and pearl millet rhizosphere samples, suggesting limited separation of bacterial communities between the two crop types. Random Forest analysis identified several taxa contributing to discrimination between sorghum and pearl millet rhizosphere samples, including members of Chloroflexi, Balneimonas, Ellin6075, TM7, and Solibacterales. However, the importance values were generally low, indicating limited discriminatory power between the two crop rhizospheres, which is consistent with the PERMANOVA results showing no significant crop-type effect on overall microbial community composition.

3. Reviewer’s comment

The manuscript frequently cites "geographic variation" between the two provinces, but the reader has no way to visualize or verify this. Include a map of the 16 sampling sites across Mpumalanga and Limpopo as a main figure. This helps clarify the spatial scale of your study.

Provide a summary meteorological data as table (or include in the site description) of the mean annual precipitation (MAP) and temperature for the sampling period. Soil microbes are highly sensitive to moisture and temperature; without this data, "geographic variation" remains an undefined variable.

Author's response:

Thank you for pointing this out. A site description including sampling period, temperature and annual precipitation has been added under site and soil collection sub-heading, under method and material.

4. Reviewer’s comment

The Biolog results show variations in carbon source utilization (e.g., carbohydrates vs. carboxylic acids). Link these functional shifts back to the soil chemistry. For instance, does higher nitrate availability correlate with the utilization of specific amino acids? Linking the Chemical (Soil), Biological (16S), and Functional (Biolog) datasets more tightly will provide a more holistic view of the rhizosphere ecosystem.

Author’s response: Thank you for the comment: We have incorporated a correlation analysis into the study to support and tighten our discussion on rhizosphere ecosystem. This analysis is included in result and discussion section.

5. Reviewers comment

Please provide a brief description of the farming systems. Were these subsistence farms? Were they under conventional tillage or conservation agriculture? This context is vital for interpreting the "intensive farming" claims made in your introduction. Your models explain a specific percentage of the variance (e.g., 7.38%). Please discuss what might account for the "unexplained" variance (e.g., unmeasured factors like soil moisture at sampling or pesticide history).

The technical foundation of your revision is strong. By adding the geographic and agronomic context requested above, you will ensure that your research provides a meaningful contribution to our understanding of C4 crop-microbe interactions in South African smallholder systems. I look forward to seeing these improvements in the next version.

Author’s response: Thank you for the comment: We have added the geographic and agronomic context in the manuscript and the unexplained variance has been discussed.

Attachment Submitted filename: Response to Reviewers_R2.docx

# Peer review: Decision Letter 2

**DOI:** 10.1371/journal.pone.0347776.r005

**Authors:** Ying Ma

8 Apr 2026

16S rRNA based metagenomics insight into the microbial diversity and functional attributes of soils from the rhizosphere of selected C4 crops of farms in Mpumalanga and Limpopo provinces, South Africa

PONE-D-25-48417R2

Dear Dr. Hassen,

We’re pleased to inform you that your manuscript has been judged scientifically suitable for publication and will be formally accepted for publication once it meets all outstanding technical requirements.

Within one week, you’ll receive an e-mail detailing the required amendments. When these have been addressed, you’ll receive a formal acceptance letter and your manuscript will be scheduled for publication.

An invoice will be generated when your article is formally accepted. Please note, if your institution has a publishing partnership with PLOS and your article meets the relevant criteria, all or part of your publication costs will be covered. Please make sure your user information is up-to-date by logging into Editorial Manager at Editorial Manager® and clicking the ‘Update My Information' link at the top of the page. For questions related to billing, please contact billing support.

If your institution or institutions have a press office, please notify them about your upcoming paper to help maximize its impact. If they’ll be preparing press materials, please inform our press team as soon as possible -- no later than 48 hours after receiving the formal acceptance. Your manuscript will remain under strict press embargo until 2 pm Eastern Time on the date of publication. For more information, please contact onepress@plos.org.

Kind regards,

Ying Ma, Ph.D.

Academic Editor

PLOS One

Additional Editor Comments (optional):

Reviewers' comments:

Reviewer's Responses to Questions

**Comments to the Author**

1. If the authors have adequately addressed your comments raised in a previous round of review and you feel that this manuscript is now acceptable for publication, you may indicate that here to bypass the “Comments to the Author” section, enter your conflict of interest statement in the “Confidential to Editor” section, and submit your "Accept" recommendation.

Reviewer #1: All comments have been addressed

**********

2. Is the manuscript technically sound, and do the data support the conclusions?

The manuscript must describe a technically sound piece of scientific research with data that supports the conclusions. Experiments must have been conducted rigorously, with appropriate controls, replication, and sample sizes. The conclusions must be drawn appropriately based on the data presented.

Reviewer #1: Yes

**********

3. Has the statistical analysis been performed appropriately and rigorously?

Reviewer #1: Yes

**********

4. Have the authors made all data underlying the findings in their manuscript fully available?

The PLOS Data policy requires authors to make all data underlying the findings described in their manuscript fully available without restriction, with rare exception (please refer to the Data Availability Statement in the manuscript PDF file). The data should be provided as part of the manuscript or its supporting information, or deposited to a public repository. For example, in addition to summary statistics, the data points behind means, medians and variance measures should be available. If there are restrictions on publicly sharing data—e.g. participant privacy or use of data from a third party—those must be specified.

Reviewer #1: Yes

**********

5. Is the manuscript presented in an intelligible fashion and written in standard English?

PLOS ONE does not copyedit accepted manuscripts, so the language in submitted articles must be clear, correct, and unambiguous. Any typographical or grammatical errors should be corrected at revision, so please note any specific errors here.

Reviewer #1: Yes

**********

6. Review Comments to the Author

Please use the space provided to explain your answers to the questions above. You may also include additional comments for the author, including concerns about dual publication, research ethics, or publication ethics. (Please upload your review as an attachment if it exceeds 20,000 characters)

Reviewer #1: (No Response)

**********

7. PLOS authors have the option to publish the peer review history of their article (what does this mean?). If published, this will include your full peer review and any attached files.

If you choose “no”, your identity will remain anonymous but your review may still be made public.

**Do you want your identity to be public for this peer review?**   For information about this choice, including consent withdrawal, please see our Privacy Policy.

Reviewer #1: **Yes:**  Edwinraj Esack

**********

# Editor report: Acceptance letter

**DOI:** 10.1371/journal.pone.0347776.r006

**Authors:** Ying Ma

PONE-D-25-48417R2

PLOS One

Dear Dr. Hassen,

I'm pleased to inform you that your manuscript has been deemed suitable for publication in PLOS One. Congratulations! Your manuscript is now being handed over to our production team.

At this stage, our production department will prepare your paper for publication. This includes ensuring the following:

* All references, tables, and figures are properly cited

* All relevant supporting information is included in the manuscript submission,

* There are no issues that prevent the paper from being properly typeset

You will receive further instructions from the production team, including instructions on how to review your proof when it is ready. Please keep in mind that we are working through a large volume of accepted articles, so please give us a few days to review your paper and let you know the next and final steps.

Lastly, if your institution or institutions have a press office, please let them know about your upcoming paper now to help maximize its impact. If they'll be preparing press materials, please inform our press team within the next 48 hours. Your manuscript will remain under strict press embargo until 2 pm Eastern Time on the date of publication. For more information, please contact onepress@plos.org.

You will receive an invoice from PLOS for your publication fee after your manuscript has reached the completed accept phase. If you receive an email requesting payment before acceptance or for any other service, this may be a phishing scheme. Learn how to identify phishing emails and protect your accounts at https://explore.plos.org/phishing.

If we can help with anything else, please email us at customercare@plos.org.

Thank you for submitting your work to PLOS ONE and supporting open access.

Kind regards,

PLOS ONE Editorial Office Staff

on behalf of

Dr. Ying Ma

Academic Editor

PLOS One
